# Screening strategy to identify Cas9 variants with higher HDR activity based on diphtheria toxin

**DOI:** 10.1186/s12929-025-01197-9

**Published:** 2025-12-03

**Authors:** Daisuke Matsumoto, Komari Kubota, Yu Sato, Tomoko Kato-Inui, Kiyomi Nigorikawa, Yuichiro Miyaoka, Wataru Nomura

**Affiliations:** 1 School of Pharmaceutical Sciences, Hiroshima University, 1-2-3 Kasumi Minami-Ku, Hiroshima, 734-8553 Japan; 2 Graduate School of Biomedical and Health Sciences, Hiroshima University, 1-2-3 Kasumi Minami-Ku, Hiroshima, 734-8553 Japan; 3 Regenerative Medicine Project, Tokyo Metropolitan Institute of Medical Science, Setagaya, Tokyo 156-8506 Japan; 4 Research Center for Thermotolerant Microbial Resources, Yamaguchi University, Yamaguchi, 753-8515 Japan

**Keywords:** Homology-directed repair, Diphtheria toxin, Screening

## Abstract

**Background:**

In gene therapy via genome editing, it is essential to precisely repair disease-associated gene sequences without introducing random mutations. However, achieving highly accurate genome editing remains challenging owing to the low efficiency of homology-directed repair (HDR)-mediated gene repair, which relies on template DNA. Therefore, if Cas9 mutants capable of enhancing HDR can be identified, they could enable more precise gene therapies.

**Method:**

In this research project, we developed a screening system that uses the acquisition of diphtheria toxin resistance as an indicator of HDR efficiency in human cells and EGFP disruption as an indicator of off-target effect.

**Results:**

By screening a library of SpCas9 variants with random mutations introduced into its nuclease domain, we identified a novel SpCas9 mutant with higher HDR efficiency than wild-type Cas9.

**Conclusion:**

We explored the possibility of obtaining Cas9 mutants with high HDR efficiency via this screening system.

**Graphical Abstract:**

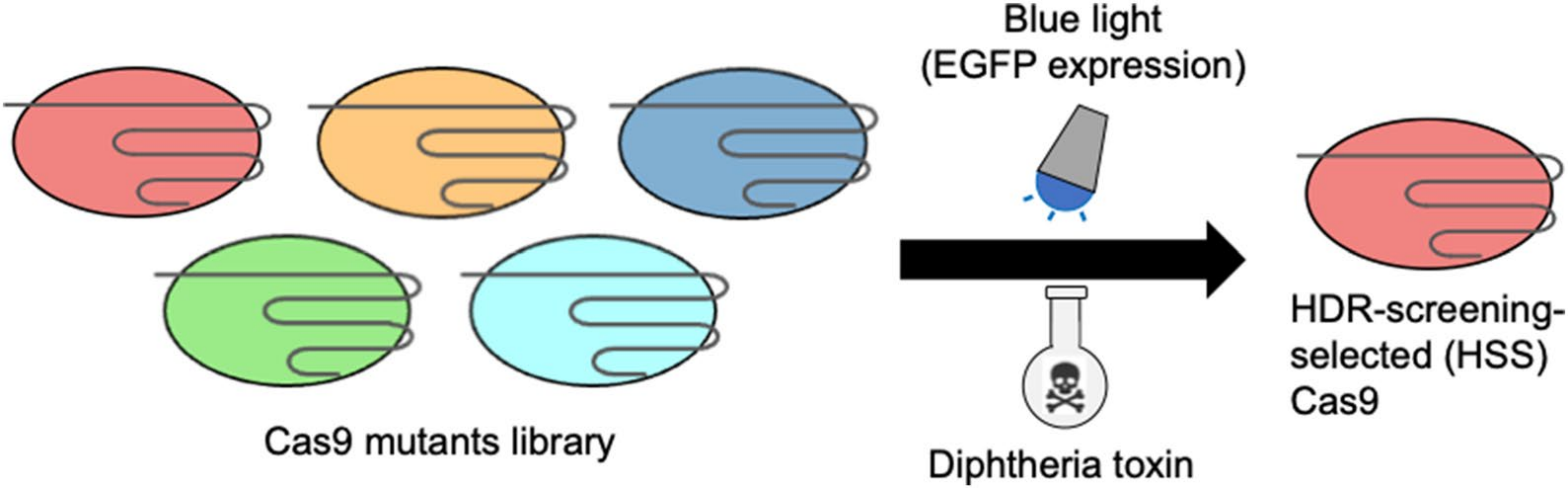

**Supplementary Information:**

The online version contains supplementary material available at 10.1186/s12929-025-01197-9.

## Background

CRISPR-Cas9 is an adaptive immune system in which bacteria have evolved to defend against invading elements such as bacteriophages [1]. This system has been harnessed for genome editing in various species, including mammals [2] and plant cells [3]. The Cas9 derived from *Streptococcus pyogenes* (SpCas9) is the first Cas9 that is applied to human cells [4]. The character of the protospacer adjacent motif (PAM) needed for Cas9 to interact with target DNA is very simple (NGG), which leads SpCas9 to be used widely. Recently, the U.S. Food and Drug Administration approved a groundbreaking therapy called Casgevy, the first CRISPR-Cas9-based drug designed for sickle cell disease treatment [5]. In addition, numerous clinical trials are currently underway to explore the potential of CRISPR-based therapies [6, 7]. However, most gene therapy applications using CRISPR-Cas9 focus on gene knockout via the nonhomologous end joining (NHEJ) repair pathway rather than precise gene correction via homology-directed repair (HDR), a pathway that repairs DNA via a template sequence. This is primarily due to the low efficiency of HDR. To expand the clinical applications of CRISPR-Cas9 beyond gene knockout, enhancing our understanding of HDR induction following Cas9-induced double-strand breaks (DSBs) and developing strategies to improve HDR efficiency are essential.

HDR is known to occur preferentially during the S/G2 phase of the cell cycle. To exploit this, we developed a CRISPR-Cas9 system that activates SpCas9 specifically during the S/G2/M phases via anti-CRISPR proteins [8, 9]. By restricting Cas9 cleavage to the S/G2 phase, we facilitated HDR after DSB induction. When we applied this system to high-fidelity SpCas9 variants, we discovered that certain SpCas9 mutants with mutations in the RuvCIII subdomain presented increased HDR efficiency particularly at target sites with low GC content [10]. Moreover, a separate study suggested that SpCas9 variants can exhibit higher HDR efficiency than can wild-type Cas9 in a target-dependent manner [11]. On the basis of these findings, we hypothesize that it is possible to engineer SpCas9 mutants that efficiently induce HDR in mammalian cells.

In this study, we designed a screening system to identify HDR-promoting Cas9 variants with minimal off-target effects. To achieve this goal, we employed a diphtheria toxin-based selection system [12] to screen for HDR-edited cells and developed an off-target detection assay based on the enhanced green fluorescent protein (EGFP) gene. After optimizing the screening conditions, we identified a novel SpCas9 mutant by screening a library of Cas9 variants with random mutations in the nuclease domain.

## Methods

### Plasmid construction for screening

All information about the oligo DNA sequences that were used in plasmid construction is shown in Supplementary Table 1. The Lenti. The SpCas9-P2A-EGFP vector was constructed via the lentiCRISPR v2 plasmid, which was a gift from the Feng Zhang laboratory (Addgene plasmid #52961) [13]. To generate this vector, the EF1a promoter was amplified via Tks Gflex^™^ DNA Polymerase (TAKARA) with the EF1a Fw primer and the EF1a Rv primer. The Cas9 and P2A genes were amplified separately via Tks Gflex^™^ DNA Polymerase (TAKARA) with the Lenti insert Cas9 Fw primer and P2A Rv primer. In addition, the GFP gene, which contains silent mutations, was constructed by assembling three DNA fragments. The first fragment was amplified via the P2A EGFP frag1 Fw primer and the P2A EGFP frag1 Rv primer. The second fragment was amplified via the P2A EGFP frag2 Fw primer and the P2A EGFP frag2 Rv primer. The third fragment was amplified via the P2A EGFP frag3 Fw primer and the P2A EGFP frag3 Rv primer. After the required gene fragments were amplified, the EF1a promoter, Cas9-P2A gene, and mutated P2A-EGFP gene were inserted into the NheI-HF (NEB) and NotI-HF (NEB)-digested HIV7-Bcl2-CD28-CD3z-T2A-EGFR plasmids. This plasmid was a gift from the David Baker laboratory (Addgene plasmid #163465) [14]. The fragments were assembled via NEBuilder HiFi DNA Assembly Master Mix (NEB). To confirm successful plasmid construction, the final construct was analyzed by Sanger sequencing.

The HBEGF-targeting sgRNA plasmid was generated from a gRNA cloning vector, which was a gift from the George Church laboratory (Addgene plasmid #41824) [15]. To construct this plasmid, the HBEGF target sgRNA Fw primer and the HBEGF target sgRNA Rv primer were annealed in Gflex reaction buffer under specific temperature conditions. The reaction was carried out at 95 °C for 5 min, followed by a gradual cooling step from 95 °C to 25 °C, decreasing at a rate of 0.1 °C per second. The reaction was then held at 4 °C. Following the annealing step, polymerase was added to the mixture, and an elongation reaction was performed at 68 °C for 2 min. The resulting DNA product was then purified via the FavorPrep GEL/PCR Purification Mini Kit (FAVORGEN). After purification, the inserted DNA was integrated into the AflII (NEB)-digested gRNA cloning vector via Gibson assembly.

To generate the EGFP off-target sgRNA library, a gRNA cloning vector was modified by introducing a six-base deletion. This deletion was introduced to remove the SnaBI recognition site, which was achieved by amplifying the entire vector via the SnaBI removal Fw primer and the SnaBI removal Rv primer. The amplified vector was then self-assembled via Gibson assembly. After the gRNA vector was modified, the constructed guide RNA plasmid was digested with AflII (NEB). The inserted DNA for the sgRNA library was prepared via two different primer sets. The EGFP off-library Fw primer and the EGFP_sgRNA_Rv primer were used for the off-target sgRNA library, whereas the EGFP on-target Fw primer and the EGFP_sgRNA_Rv primer were used for the on-target sgRNA. For the off-target sgRNA library, 100 ng of AflII-treated vector DNA was combined with 10 ng of the library insert DNA and assembled via Gibson assembly. The assembled product was then purified via a MicroElute GEL/PCR Clean-Up Column, with an elution volume of 6 μL. Following purification, the assembled sgRNA library DNA was transformed into NEB stable competent cells (NEB) via a Gene Pulser (Bio-Rad) for electroporation. The transformed cells were immediately recovered and cultured in 50 mL of LB medium supplemented with 50 μg/mL kanamycin. The bacterial culture was incubated at 37 °C overnight to allow for plasmid replication and expansion.

### Toxicity assay of diphtheria toxin

hTERT-RPE1 cells were cultured in DMEM/F12 medium (FUJIFILM Wako) supplemented with 10% fetal bovine serum (FBS) and 1% penicillin–streptomycin (FUJIFILM Wako). For initial optimization of the seeding cell number, hTERT-RPE1 cells were seeded in a 96-well plate using a two-fold serial dilution starting from 25,000 cells per well. The next day, the number of viable cells was measured using the Cell Counting Kit-8 (DOJINDO) according to the manufacturer’s instructions. To assess cell death induced by diphtheria toxin (DT), hTERT-RPE1 cells were seeded at 4 × 10^3^ cells per well in a 96-well plate and cultured overnight. DT (FUJIFILM Wako) was diluted to 20 μg/mL in PBS from the original stock solution. The DT-containing medium was then added to the wells to achieve a final DT concentration of 20 ng/mL in a total volume of 200 μL per well. For the negative and positive control samples, medium without DT was used. Three days after DT treatment, cytotoxicity was assessed using the Cell Counting Kit-8 (DOJINDO), following the manufacturer’s instructions.

### Error-prone PCR and library construction

Error-prone PCR was performed via the Diversify™ PCR Random Mutagenesis Kit (TAKARA) according to the manufacturer’s protocol. The concentrations of MnSO₄ and dGTP were adjusted depending on the desired mutation frequency, as higher concentrations of these reagents increase the mutation rate. The Lenti. The SpCas9-P2A-EGFP plasmid was used as the template DNA for error-prone PCR. The amplification was carried out via the nuclease domain mutation Fw primer and the nuclease domain mutation Rv primer. The reaction conditions included an initial denaturation step at 94 °C for 30 s, followed by 25 cycles of 94 °C for 30 s for denaturation and 68 °C for 1.5 min for amplification. A final extension step was performed at 68 °C for 1 min, and the reaction was then held at 4 °C. After amplification, the PCR products were subjected to 1% agarose gel electrophoresis to confirm the presence of the expected DNA fragments. The target DNA bands were excised from the gel, and the DNA was purified via a standard gel extraction method. The purified DNA fragments were then inserted into the Lenti. The SpCas9-P2A-EGFP vector was preamplified via KOD One Master Mix (TOYOBO). The Gibson assembly method was used to insert the mutated DNA fragments into the vector. The reaction was performed with the PI Fw primer and the RuvCII Rv primer to ensure the correct incorporation of the modified sequences. Once the assembly was complete, the mutant Cas9 plasmid library was transformed into NEB stable competent cells via electroporation. The electroporation process was conducted via a MicroPulser (Bio-Rad) with a 0.2 cm gap cuvette under Ec1 electroporation conditions. Following electroporation, the transformed cells were recovered in SOC medium and incubated at 32 °C for 1 h to allow initial bacterial growth. After the recovery period, the transformed bacterial culture was transferred to 50 mL of LB medium supplemented with 100 μg/mL ampicillin. The culture was incubated at 32 °C for 18 h to ensure sufficient plasmid replication and expansion of the mutant library. Following the incubation period, plasmid DNA was extracted from the bacterial culture via the GenElute^™^ Plasmid Miniprep Kit (Sigma). The purified mutant Cas9 plasmid library was then stored for further analysis and screening in subsequent experiments.

### Lentiviral construction

HEK293T cells were seeded in 10 cm culture dishes to achieve optimal confluency for transfection. A mixture of the Lenti. The SpCas9 (library)-P2A-EGFP plasmid, the psPAX2 plasmid, and the pMD2. G plasmids were prepared at a ratio of 4:3:1. Transfection was performed via Lipofectamine 3000 (Thermo Fisher Scientific) according to the manufacturer’s instructions. The transfection mixture was scaled up to account for the larger surface area of the 10 cm dishes, which is equivalent to four times the transfection volume typically used for a 6-well plate. The supernatant, containing the lentiviral particles, was collected at 48 h and 72 h posttransfection. After each collection, the supernatant was mixed with a Lenti-X concentrator (TAKARA) and stored at 4 °C overnight to allow for the precipitation of virus particles. The virus particles were subsequently collected by centrifugation at 1500 × g for 45 min at 4 °C. Following centrifugation, the supernatant was carefully removed, and the virus particles were resuspended in PBS at a volume corresponding to 1/100th of the total culture medium volume.

For screening, hTERT-RPE-1 cells were selected as the target cells for lentiviral infection. These cells were seeded in T75 flasks and cultured in DMEM/F12 medium (FUJIFILM Wako) supplemented with 10% fetal bovine serum (FBS) and 1% penicillin‒streptomycin (FUJIFILM Wako). Prior to infection, the culture medium was replaced with fresh medium containing 8 μg/mL polybrene (Nacalai Tesque) to increase viral transduction efficiency. The prepared lentivirus mixture was added to flasks containing hTERT-RPE1 cells. The volume of the virus mixture was adjusted so that the ratio of virus particles to cell number would yield approximately 30–50% EGFP-positive cells, as determined by a titer assay conducted on a 12-well plate. After 48 h of incubation to allow for virus integration, the infected cells were sorted on the basis of EGFP positivity via a SORP Aria cell sorter (BD Biosciences). This sorting step ensured the enrichment of successfully transduced cells for further analysis.

### Screening and mutation detection

EGFP-positive hTERT-RPE1 cells were cultured for more than 48 h in DMEM/F12 medium (FUJIFILM Wako) supplemented with 10% fetal bovine serum (FBS) and 1% penicillin‒streptomycin (FUJIFILM Wako). This step allowed the cells to recover after sorting before further experiments were performed. For the transfection process, 1.5 μg of hHBEGF-targeting sgRNA plasmid, 2.5 μg of template DNA, and 5 μg of GFP off-target plasmid were introduced into 1 × 10⁶ EGFP-positive hTERT-RPE1 cells. Electroporation was performed via the Neon^®^ Transfection System (Thermo Fisher Scientific) with a 100 μL tip under the following pulse conditions: 1100 V, 20 ms, and 2 pulses. To achieve a sufficient number of transfected cells, the electroporation process was repeated five times, resulting in a total of 5 × 10⁶ transfected cells. At 48 h posttransfection, 20 ng/mL diphtheria toxin was added to selectively eliminate cells that had not undergone HDR. The selection process was carried out over a period of five days to ensure enrichment of successfully edited cells.

Following the selection period, genomic DNA was extracted from the surviving cells via ISOGENOME (NIPPON GENE) according to the manufacturer’s protocol. The mutation site was amplified via KOD One Master Mix (TOYOBO). The amplified fragment was then inserted into the Lenti. The SpCas9-P2A-EGFP vector was preamplified via KOD One Master Mix. The assembly of the mutated sequence into the vector was performed via Gibson assembly, after which the PI Fw primer and the RuvCII Rv primer were used to ensure precise integration. The assembled mutant Cas9 plasmid library was subsequently transformed into NEB stable competent cells via heat shock transformation. The transformed bacterial cells were spread onto LB agar plates containing 100 μg/mL ampicillin to allow the selection of successful transformants. Individual colonies were picked and cultured in LB medium supplemented with 100 μg/mL ampicillin for plasmid expansion. After plasmid purification, the mutation sites were confirmed by Sanger sequencing, ensuring that the desired modifications were successfully introduced.

### *Cas9 recombinant protein purification and *in vitro* Cas9 cleavage*

The SpCas9 gene was amplified from the pCas9 plasmid, which was a gift from Luciano Marraffini (Addgene #42876) [16], via KOD One polymerase (TOYOBO). Amplification was performed via the primers SpCas9_gibF and SpCas9_gibR. Simultaneously, the pET28 vector was amplified using pET_gibF and pET_gibR. The amplified fragments were then assembled via the Gibson assembly method to generate the complete expression construct. For the I795V/K918E Cas9 mutant, two mutations were introduced via PCR-based site-directed mutagenesis. The mutations were incorporated via the primers SpCas9mut_gibF and SpCas9mut_gibR for the Cas9 gene and pETmut_gibF and pETmut_gibR for the vector. The mutated fragments were assembled via Gibson assembly, and the recombinant plasmid was transformed into TOP10 competent cells (Invitrogen). The presence of the I795V and K918E mutations was confirmed by Sanger sequencing. The pET28a-SpCas9 or pET28a-SpCas9 (I795V/K918E mutant) plasmid was introduced into *Escherichia coli* Rosetta2 (DE3) pLysS via heat shock transformation. The transformation was carried out at 42 °C for 45 s, followed by recovery in SOC medium. The transformed cells were precultured overnight at 37 °C with shaking at 180 rpm in LB broth supplemented with 50 μg/mL kanamycin and 17 μg/mL chloramphenicol. After overnight preculture, 500 μL of the culture was transferred into 50 mL of fresh LB medium containing the same antibiotics in a 500 mL flask. The culture was incubated at 30 °C with shaking at 180 rpm until the early logarithmic phase (OD600 = 0.2–0.4) was reached. At this stage, isopropyl-β-D-thiogalactopyranoside (IPTG) was added to a final concentration of 0.2 mM to induce Cas9 protein expression. The induction was carried out at 30 °C for 12 h. After induction, the bacterial cells were harvested and washed twice with 50 mM Tris–HCl (pH 7.5). The cells were then resuspended in the same buffer at a concentration of 100 mg wet cells/mL. Cell lysis was performed via an ultrasonic disruptor (SONICSTAR 85, As One Corp.) to release the recombinant Cas9 protein. The crude lysate was centrifuged at 15,000 × g for 10 min at 4 °C, and the soluble fraction was separated from the pellet. The pellet was washed twice with 50 mM Tris–HCl (pH 7.5) and then resuspended in an equal volume of the same buffer to obtain the insoluble fraction. Purification of His-tagged Cas9 proteins was performed via the His-Spin Protein Miniprep Kit (Zymo Research) according to the manufacturer’s protocol. For SDS‒PAGE analysis, 5 μL of each fraction (insoluble, soluble, and His-tag purified) was loaded onto the gel. The gel was stained with Coomassie Brilliant Blue R250 to visualize the protein bands.

To evaluate the DNA cleavage activity of the recombinant wild-type and mutant SpCas9 proteins, in vitro cleavage assays were performed using AAVS1- and VEGFA-targeting sgRNAs. The sgRNAs were transcribed in vitro via the T7 Transcription Kit (TAKARA). The sgRNA templates were amplified from a sgRNA expression vector via the primers IVT AAVS1 sgRNA Fw or IVT VEGFA sgRNA Fw, along with IVT polyT Rv primers. After DNase treatment, the transcribed sgRNAs were purified via phenol/chloroform/isoamyl alcohol extraction (25:24:1) (NIPPON GENE), followed by ethanol precipitation. The purified sgRNAs were dissolved in UltraPure™ DNase/RNase-Free distilled water (Invitrogen) for subsequent experiments. For the cleavage assay, 20 ng of recombinant Cas9 protein and 20 ng of in vitro-transcribed sgRNA were mixed in NEBuffer 3.1 (NEB) and incubated at room temperature for 10 min to allow Cas9–sgRNA complex formation. A 9 μL aliquot of the mixture was then added to 1 μL of substrate DNA (150 ng/μL). The cleavage reaction was carried out for 1, 2, 3, or 4 h at 37 °C. The reaction was stopped by adding proteinase K (Nacalai Tesque), followed by incubation at 56 °C for 30 min to degrade the protein components. The cleaved DNA fragments were analyzed via MultiNA (Shimadzu), an automated microchip electrophoresis system, along with dedicated MultiNA software to determine cleavage efficiency.

### Plasmid construction for confirmed Cas9 mutant activity

The genes of Cas9 mutants with relatively high activity (#13, #24, and #27) were amplified via Gflex polymerase (TAKARA) and inserted into a NotI-digested pEBMulti-Hyg episomal vector (FUJIFILM Wako) via Gibson assembly. The assembled plasmids were then transformed into NEB stable competent cells, and transformants were selected via the use of 100 μg/mL hygromycin (FUJIFILM Wako). The presence of mutations in each mutant was confirmed by Sanger sequencing. Each mutant in the pEBMulti-Hyg vector was subsequently used for an ICE analysis assay.

For the ddPCR assay, the Cas9 expression vector was switched from pEBMulti-Hyg to px330, a vector previously reported in the literature [10]. To achieve this goal, we constructed a pGB BsmBI cassette vector derived from the pGB vector (Plasmid #204744, Addgene) [17]. First, the sgRNA scaffold DNA was generated by annealing the sgRNA scaffold Fw and sgRNA scaffold Rv oligonucleotides, followed by elongation via Phusion polymerase (NEB). Three separate DNA fragments were amplified from the pGB vector via Gflex polymerase (TAKARA) with different primer combinations. The first fragment was amplified via the pGB vector Fw, and the Rv primers were removed from BsmBI. The second fragment was generated via the removal of the Fw and pGB vector Rv primers from BsmBI, whereas the third fragment was amplified via the pGB Insert Fw and pGB Insert Rv primers. These three fragments were assembled via Gibson assembly to reconstruct the vector. After assembly, the pGB BsmBI cassette vector was digested with SacI (TAKARA). To insert the internal ribosome entry site type 2 (IRES2) sequence, we amplified it from pIRES2-DsRed-Express2 (TAKARA) via the IRES2 Fw and IRES2 Rv primers. Additionally, the puromycin resistance gene was amplified from lentiCRISPR v2 (Addgene #52961) via the puro Fw and puro Rv primers. These two fragments were then inserted into the SacI-digested pGB BsmBI cassette vector, and the resulting plasmid was designated “pGB BsmBI cassette IRES2-Puro.” To insert wild-type Cas9 or Cas9 variants, including mutant #27, with or without AcrIIA4-Cdt1, we digested the pGB BsmBI cassette IRES2-Puro vector with AgeI and EcoRI, followed by Gibson assembly. In the case of constructs encoding wild-type SpCas9 or Cas9 variants alone, the corresponding Cas9 fragments were amplified via the GA A4C/Cas9 Fw and GA Cas9 Rv primers. For constructs encoding AcrIIA4-Cdt1-T2A-SpCas9, the AcrIIA4-Cdt1-T2A fragment was amplified via the GA A4C/SpCas9 Fw and T2A Rv primers, and the T2A-SpCas9 fragment was amplified via the T2A SpCas9 Fw + GA SpCas9 Rv primers. For constructs encoding Cas9-T2A (or P2A)-AcrIIA4-Cdt1, Cas9-T2A (or -P2A) fragment was amplified via the GA Cas9 Fw and T2A (or P2A) Cas9 Rv primers. T2A-(or P2A-) AcrIIA4-Cdt1 fragment was amplified via the T2A (or P2A) A4C Fw and GA A4C Rv for px330 primers. All PCRs were performed via KOD One polymerase (TOYOBO).

To generate an IRES2-mediated AcrIIA4-Cdt1 expression vector, the pGB BsmBI cassette vector was modified. The AcrIIA4-Cdt1 gene was amplified via KOD One polymerase (TOYOBO) with the AcrIIA4 Fw and Cdt1 Rv primers. The vector backbone, excluding the puromycin resistance gene, was amplified via Gflex polymerase (TAKARA) with the IRES2 Rv and bGH primers. These two fragments were assembled via Gibson assembly. The Cbh promoter was replaced with the original promoter from the pGB BsmBI cassette vector by digestion with FastDigest PstI (Thermo Fisher Scientific) and BshTI (AgeI) (Thermo Fisher Scientific). This step was followed by ligation via Ligation High Ver.2 (TOYOBO). The newly constructed vector was designated the “pGB BsmBI cassette IRES2-AcrIIA4-Cdt1 vector.” For the FMDVIRES-AcrIIA4-Cdt1 construct, FMDV IRES sequence was amplified via FMDV IRES Fw and FMDV IRES Rv primers from PB-IM-ERN-hMyoD1 (FMDV IRES) vector gifted from Dr. Hidetoshi Sakurai. Then, EcoRI and BalI treated fragment and pGB BsmBI cassette IRES2-AcrIIA4-Cdt1 vector were ligated. The EGFP-T2A-puromycin resistance gene was then amplified via KOD One polymerase (TOYOBO) with EGFP Fw for IRES2 A4C and Puro Rv for IRES2 A4C primers. The amplified fragment was inserted into AgeI/EcoRI-digested pGB BsmBI cassette IRES2 (or FMDVIRES)-AcrIIA4-Cdt1 vectors via Gibson assembly. The EGFP gene was subsequently removed by BamHI and AflII digestion, and wild-type Cas9 or the acquired Cas9 mutant gene (amplified with KOD One polymerase using GA Cas9 Fw and GA Cas9 T2A Rv primers) was inserted via Gibson assembly.

### ICE analysis for HDR, target and off-target mutation detection

HEK293A and HeLa cells were cultured in Dulbecco’s modified Eagle’s medium (DMEM) supplemented with 10% fetal bovine serum (FBS) and 1% penicillin‒streptomycin. Once the cells reached an appropriate confluence, they were seeded into 6-well plates for transfection. Each well of cells was transfected with a pEBMulti-Hyg episomal vector encoding either wild-type SpCas9 or the acquired SpCas9 mutant #27. The transfection was performed via Lipofectamine 3000 (Thermo Fisher Scientific) following the manufacturer’s instructions. The next day, the transfected cells were transferred to 6 cm culture dishes and subjected to antibiotic selection with 350 μg/mL hygromycin B (FUJIFILM Wako). The cells were maintained under selection for three days to ensure the elimination of untransfected cells. After the selection period, the surviving cells were further transfected with 150 ng of plasmid DNA encoding sgRNA and 25 pmol of single-stranded oligodeoxynucleotide (ssODN) as a donor template for HDR. This donor DNA had a 60 bp homology arm and introduced a 9-base insertion containing a HindIII recognition site at the target site after HDR. The transfection was carried out via the Neon^®^ Transfection System (Thermo Fisher Scientific), which facilitates efficient delivery of nucleic acids via electroporation. The electroporation conditions were optimized for each cell type to ensure maximal transfection efficiency. HEK293A cells were electroporated at 1245 V for 10 ms with three pulses, whereas HeLa cells were electroporated at 1005 V for 35 ms with two pulses. Following electroporation, the cells were cultured for 72 h to allow sufficient time for HDR-mediated genome editing to occur. After the incubation period, genomic DNA was extracted from the cells via ISOGENOME (NIPPON GENE) according to the manufacturer’s protocol. The genomic regions of interest, including both on-target and off-target sites, were amplified via PCR via Gflex polymerase (TAKARA) to assess editing efficiency.

The PCRs were performed under the following cycling conditions. First, an initial denaturation step was carried out at 94 °C for 2 min. This was followed by 10 cycles of preamplification, consisting of 98 °C for 10 s, 68 °C to 63.5 °C with a decrease of 0.5 °C per cycle for 15 s, and 68 °C for 30 s. The main amplification phase consisted of 25 cycles of 98 °C for 10 s, 63 °C for 15 s, and 68 °C for 30 s. A final extension step was performed at 68 °C for 1 min. Following amplification, the PCR products were purified via the QIAquick PCR Purification Kit (Qiagen). A total of 100 ng of purified DNA was prepared for Sanger sequencing via specific primers. The sequencing was performed by Eurofins Genomics. The obtained sequence data were analyzed via ICE analysis software (Synthego), which enables quantification of editing efficiency and assessment of mutation profiles at the target sites. Finally, all the statistical analyses were performed via R Studio to evaluate the significance of the observed editing events.

### Cell cycle analysis to assess the effect of HSS Cas9 on cell cycle progression

HEK293A cells were cultured in Dulbecco’s Modified Eagle’s Medium (DMEM) supplemented with 10% fetal bovine serum (FBS) and 1% penicillin–streptomycin. The cells were seeded into 12-well plates at a density of 2 × 10^5^ cells per well. The cells in each well were transfected with a pGB BsmBI cassette vector in one of the following forms: an empty vector, a vector encoding wild-type (WT) Cas9 and an ATP7B-targeting sgRNA, or a vector encoding HSS Cas9 and the same sgRNA, using Lipofectamine 3000 (Thermo Fisher Scientific). After 16 h of incubation, the medium was replaced with fresh medium containing 2.5 μg/mL puromycin (FUJIFILM Wako) to select transfected cells. Following 48 h of selection, the cells were stained with 5 μg/mL Hoechst 33342 in medium for 30 min in a CO₂ incubator. The stained cells were then collected using trypsin–EDTA and DMEM (both contain 5 μg/mL Hoechst 33342) and filtered through a cell strainer (FALCON). The filtered cells were analyzed using an LSRFortessa™ X-20 flow cytometer (BD Biosciences).

### Droplet digital PCR (ddPCR) analysis for HDR and indels detection

HEK293A cells were cultured in Dulbecco’s modified Eagle’s medium (DMEM) supplemented with 10% fetal bovine serum (FBS) and 1% penicillin‒streptomycin. Ten thousand cells were seeded into a 96-well plate. For transfection, each well of cells was transfected with a pGC vector targeting the ATP7B, GRN, or RBM20 genes, along with a single-stranded oligodeoxynucleotide (ssODN) as the donor template. This donor DNA had a 60 bp homology arm and introduced a 1-base substitution at the target site after HDR. Transfection was performed via Lipofectamine 3000 (Thermo Fisher Scientific) according to the manufacturer’s protocol. After 16 h of incubation, the culture medium was replaced with fresh medium containing 2.5 μg/mL puromycin (FUJIFILM Wako) to select for successfully transfected cells. The cells were maintained in puromycin-containing medium for 72 h to ensure the elimination of untransfected cells.

The detailed protocols for genomic DNA extraction are described in ref 10. In brief, genomic DNA was extracted via cell lysis and ethanol precipitation. Thirty microlitres of TE buffer was added to each well. The DNA concentration was measured via a DS-11 spectrophotometer (DeNovix) to ensure adequate sample quantity for downstream analysis.

The digital PCR assay used to detect HDR and indels activities followed a previously described protocol [18]. To prepare samples for droplet digital PCR (ddPCR), 100–180 ng of genomic DNA was mixed with 12 μL of 2 × ddPCR Supermix for Probes (no dUTP) (Bio-Rad Laboratories, Hercules, CA, USA). Additionally, 0.6 μL of primers and probe sets (as detailed in Table S5, reference [11]) and 0.48 μL of restriction enzyme were added to fragment the DNA. The HindIII enzyme was used for ATP7B and RBM20, while RspRSII was used for GRN. The final volume of the reaction mixture was adjusted to 24 μL with nuclease-free water. To generate nanoliter-scale droplets containing the PCR reagents, the reaction mixtures were processed via a droplet generator (Bio-Rad Laboratories). The generated droplets were transferred to an Eppendorf twin.tec PCR plate (Eppendorf), which was subsequently sealed via a PCR plate heat sealer (Bio-Rad Laboratories) and a PX1 PCR plate sealer (Bio-Rad), following the manufacturer’s guidelines. Thermal cycling was performed using a C1000 Touch Thermal Cycler (Bio-Rad). After PCR amplification, the fluorescence signals of the droplets were analyzed via a QX200 Droplet Reader (Bio-Rad). This ddPCR assay was specifically designed to detect three different alleles: the wild-type allele, the HDR allele, and the indels allele. The fluorescence detection system classified droplets as FAM and HEX double-positive for HDR events, FAM highly positive for wild-type alleles, and FAM single-positive for indels events (as detailed in Figure S2 in reference [11]). The frequency of HDR and indels alleles was calculated from the ddPCR data and visualized via superimposed bar graphs and dot plots. The components used for the digital PCR assay are listed in Table S5, reference (11), while the thermal cycling conditions for ddPCR are detailed in Table S6, reference (11).

### Statistics

Statistical analysis was performed using R. Multiple comparisons among groups were conducted using Tukey’s Honestly Significant Difference (HSD) test. The assumptions of the Tukey test included: independence of observations, approximate normal distribution of the populations, and homogeneity of variances across groups. Statistical differences between two groups were assessed using a non-paired 2-tailed Student’s t-test, assuming equal variances.

## Results

### Optimization of screening conditions

**Fig. 1:**
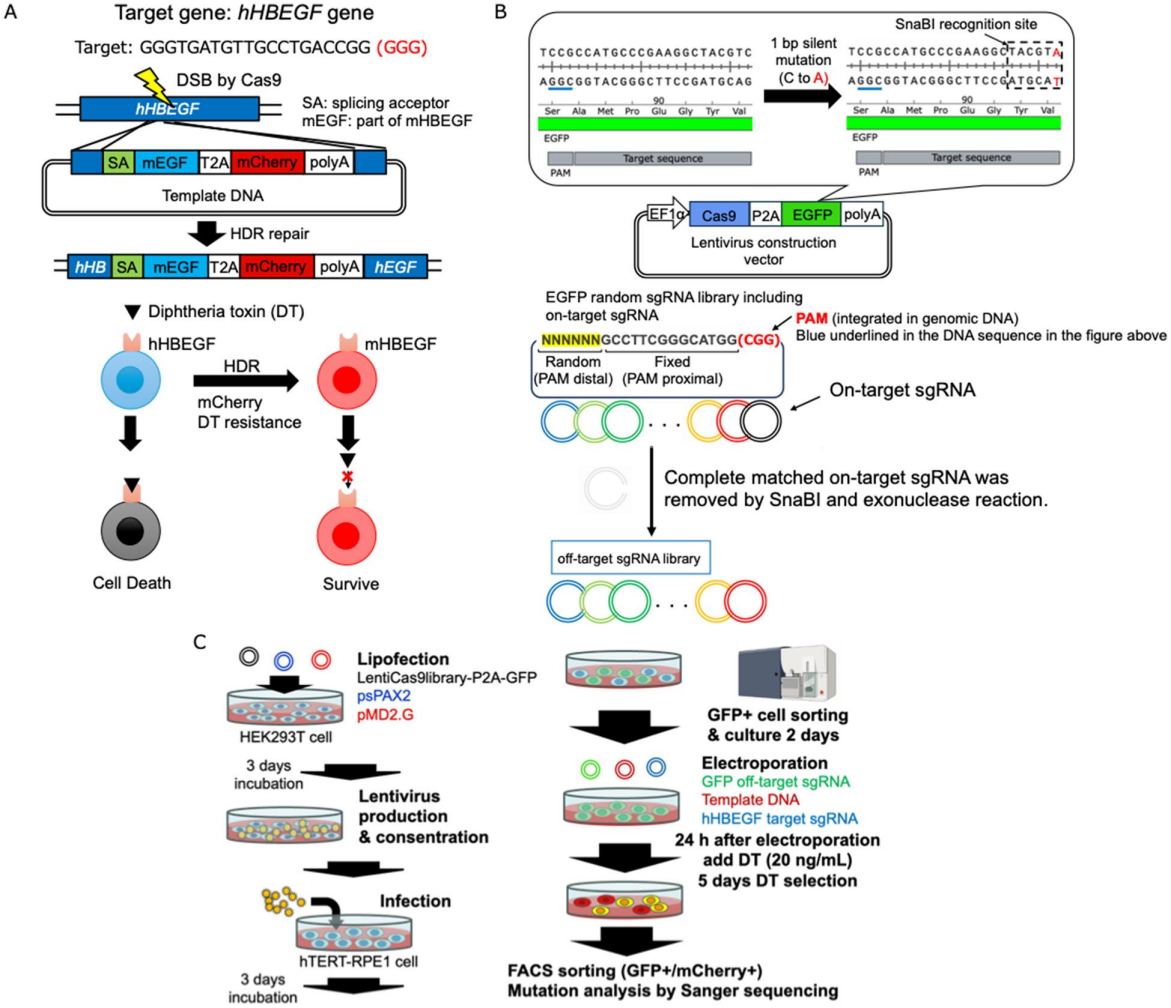
Scheme of the HDR-inducible Cas9 screening system. **A** HDR occurred during cell selection by Diphtheria toxin (DT). The human HBEGF (hHBEGF) gene was edited into a E141K mutated hHBEGF (mHBEGF) downstream of the cleavage site through HDR repair [12]. Then, human cells acquire DT resistance. **B** Off-target effects of EGFP expression on the cell selection system. The PAM sequence of the target site in the EGFP gene is underlined with a blue line. A silent mutation was introduced into the 5′ end of the EGFP target site to insert the SnaBI recognition site. A library of off-target sgRNAs targeting randomly mutated sequences 6 bases from the 5′ end was constructed by removing the target site recognition sgRNA plasmid via SnaBI and exonuclease. **C** A scheme for the selection of HDR-inducible Cas9 from a mutant library (**C**) was created with BioRender.com

To identify Cas9 mutants that promote HDR while maintaining low off-target activity, we developed a screening system based on a Cas9 mutant library. The screening method utilized diphtheria toxin (DT) as an indicator of HDR occurrence (Fig. 1A). DT selectively binds to human HBEGF (hHBEGF) and enters cells via endocytosis. Once inside, DT inhibits elongation factor-2, leading to protein synthesis arrest and subsequent death in human cells. However, DT does not bind to E141K mutated hHBEGF (mHBEGF) [12]. Therefore, human cells in which the hHBEGF gene was successfully converted to mHBEGF via HDR were expected to survive DT selection. To facilitate this approach, we constructed template DNA and an hHBEGF-targeting sgRNA plasmid on the basis of sequences from a previous study [12]. To assess off-target effects, we used the EGFP gene (Fig. 1B). A silent mutation was introduced at V93 of the EGFP gene, creating a SnaBI restriction site. The sgRNA was designed so that the silent mutation site was located at the 5′ end of the target sequence. Since previous studies [19, 20] indicate that mutations located further from the PAM sequence are more prone to cleavage by Cas9, we constructed a sgRNA library containing random mutations at six bases on the 5′ end of the sgRNA. After the EGFP-targeting sgRNA library plasmid was constructed, the plasmid DNA encoding the sgRNA that fully matched the on-target sequence was selectively digested via SnaBI and exonuclease treatment. This process allowed us to construct an EGFP off-target sgRNA library containing mismatches ranging from one to six bases from the on-target sequence, which enabled the assessment of Cas9 tolerance to mismatches. If EGFP disruption occurred via these off-target sgRNAs, the corresponding Cas9 mutant was considered more tolerant of mismatches, indicating potential off-target activity. The screening scheme is illustrated in Fig. 1C. First, Cas9 mutant genes were delivered into hTERT-RPE1 cells via lentiviral transduction. EGFP-positive cells, which also expressed a Cas9 mutant, were sorted via fluorescence-activated cell sorting (FACS). The EGFP off-target sgRNA library plasmid, hHBEGF-targeting sgRNA plasmid, and template DNA plasmid were then introduced via electroporation. After five days of DT selection, the mutation sites in the nuclease domain of Cas9 were identified via Sanger sequencing.

**Fig. 2:**
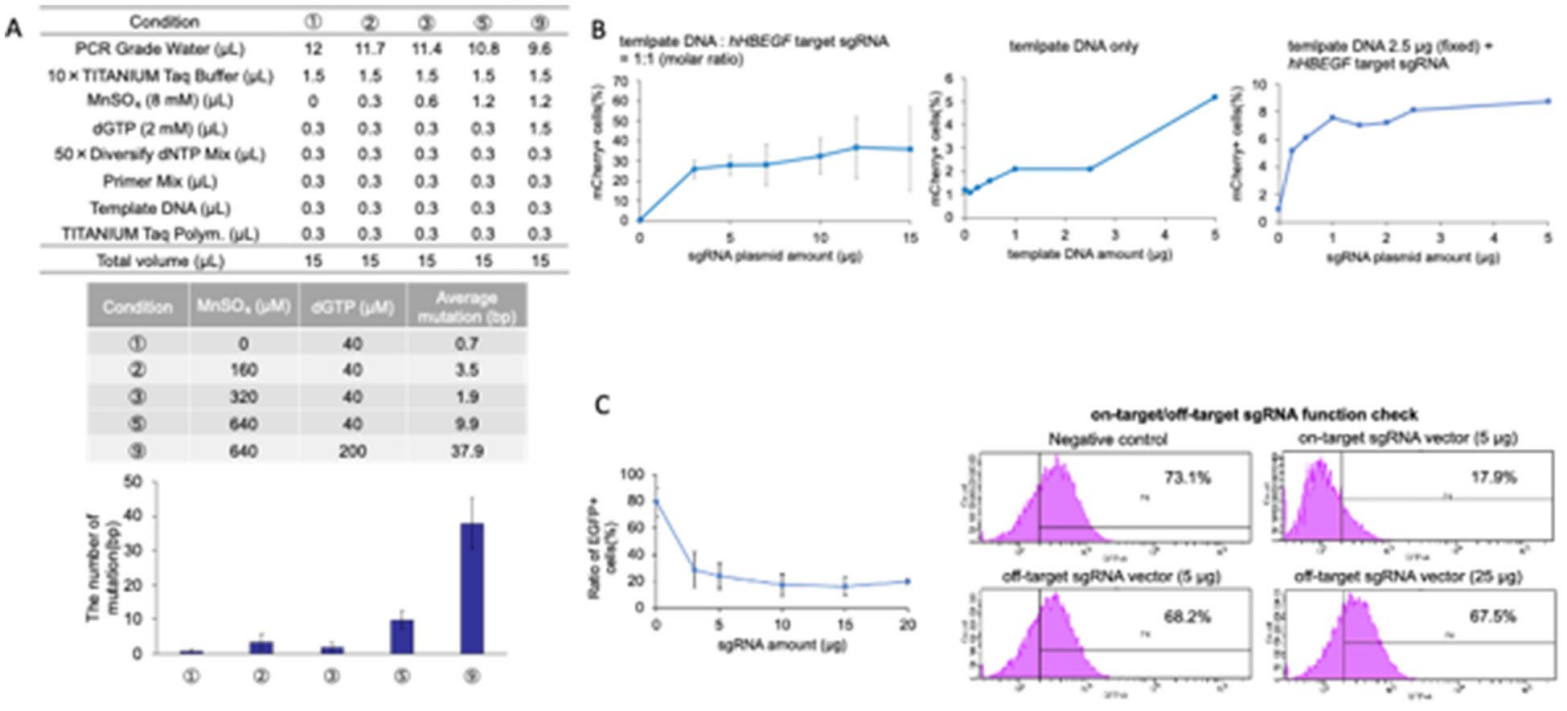
Optimization of screening conditions. **A** Consideration of the conditions for introducing a random mutation in the nuclease domain of SpCas9 via a Diversify PCR Random Mutagenesis Kit. **B** Optimization of the amount of template DNA and hHBEGF target sgRNA for screening of hTERT-RPE1 cells via the Neon Transfection System 100 μL kit. The pulse conditions were 1100 V, 20 ms, and two pulses. The left line graph shows the ratio of mCherry-positive cells when the amount of template DNA and sgRNA-encoding plasmid was increased while the molar ratio was maintained at 1:1. The middle line graph shows the ratio of mCherry-positive cells when only template DNA was transfected into hTERT-RPE-1 cells. The right line graph shows the ratio of mCherry-positive cells when the amount of hHBEGF target sgRNA was increased while the amount of template DNA was maintained at 2.5 μg. **C** Consideration of the amount of the EGFP off-target sgRNA library. The left line graph shows the ratio of EGFP-positive cells when the amount of EGFP target sgRNA was increased. The right date of flow cytometry shows the ratio of EGFP-positive cells when the off-target sgRNA library was introduced into hTERT-RPE1 cells. A wild-type Cas9-encoding lentiviral vector was used, as shown in Fig. 1B and C

To construct the Cas9 mutant library, we optimized error-prone PCR conditions. Five different conditions from the manufacturer’s manual were tested. DNA fragments spanning the RuvCII to RuvCIII subdomains were amplified in an error prone manner. Among the tested conditions, Condition No. 3 presented no wild-type sequences and contained one to four mutations per clone across the 10 analyzed clones (Fig. 2A). Therefore, Condition No. 3 was selected for Cas9 mutant library generation. We next optimized the concentration of hHBEGF-targeting sgRNA plasmid DNA and template DNA to increase HDR efficiency. The total amount of plasmid DNA used was 38.5 μg, consisting of 15 μg of sgRNA plasmid DNA and 23.5 μg of template DNA in a 1:1 molar ratio. The total mixture was diluted to 12 μg, 10 μg, 7 μg, 5 μg, or 3 μg of sgRNA plasmid DNA to determine the optimal concentration. As a result, the proportion of mCherry-positive cells plateaued at 3 μg of sgRNA plasmid DNA (Fig. 2B, left). To verify whether template DNA could integrate without Cas9 cleavage, we tested different template DNA amounts. A 5 μg concentration of template DNA resulted in an increase in mCherry-positive cells (Fig. 2B, center). On the basis of these findings, we selected 2.5 μg of template DNA for further experiments. Next, we optimized the amount of hHBEGF-targeting sgRNA plasmid DNA required for efficient HDR. When 2.5 μg of template DNA was used, the proportion of mCherry-positive cells reached a plateau at 1 μg of hHBEGF-targeting sgRNA plasmid DNA (Fig. 2B, right). Therefore, we decided to use 1.5 μg of hHBEGF sgRNA plasmid DNA to maintain a 1:1 molar ratio with template DNA. To determine the optimal DT selection duration, we confirmed that HDR-repaired cells were effectively enriched after three days of DT treatment (Supplementary Fig. 1). Furthermore, the 3-day treatment with diphtheria toxin resulted in a loss of viability in most unedited cells, suggesting that this approach enables efficient HDR-edited cell selection by FACS (Supplementary Fig. 2).

Finally, we optimized the amount of EGFP off-target sgRNA plasmid DNA to detect off-target effects. We first confirmed that EGFP gene disruption increased with increasing amounts of EGFP-targeting sgRNA plasmid DNA (Fig. 2C, left). We then tested different concentrations of EGFP off-target sgRNA plasmid DNA and observed that the EGFP disruption rates were similar when 5 μg (approximately 5% decrease) and 25 μg (approximately 6% decrease) of DNA were used (Fig. 2C, right). In contrast, when 5 μg of EGFP-targeting sgRNA plasmid DNA was used, more than 50% of the cells lost EGFP expression, demonstrating the efficiency of on-target cleavage. On the basis of these results, we decided to use 5 μg of EGFP off-target sgRNA plasmid DNA for screening.

### Activity check of screened mutants

After diphtheria toxin (DT) treatment, EGFP- and mCherry-positive cells were enriched in both the wild-type Cas9 control and the mutant library samples (Supplementary Fig. 3). To identify mutations in the Cas9 gene, the mutated region was cloned and inserted into plasmid DNA, and Sanger sequencing was performed on 30 individual colonies. The mutations identified in these clones are summarized in Supplementary Fig. 4. From the sequenced clones, 11 candidates were selected on the basis of the presence of at least one mutation in the RuvCIII subdomain. This selection was based on our previous study, which suggested that mutations in the RuvCIII subdomain, such as eSpCas9, SpCas9-HF1, and LZ3 Cas9, could increase HDR repair efficiency at the EMX1 gene target site [10]. To evaluate the precise editing efficiency of each mutant, we analyzed HDR-mediated genome repair via ssODN, which introduces a 12-base insertion containing the HindIII recognition site at the AAVS1 and VEGFA target sites. Among the tested mutants, two clones (#13 and #24) demonstrated HDR-mediated repair at the AAVS1 site, whereas four clones (#2, #13, #24, and #27) presented HDR-mediated repair at the VEGFA site (Supplementary Fig. 5). The remaining clones did not exhibit HDR-mediated genome editing, indicating that their mutations did not increase HDR efficiency. On the basis of these results, we selected three promising Cas9 mutants for further analysis: clones #13 and #24, which demonstrated HDR-mediated repair at both the AAVS1 and VEGFA target sites, and clone #27, which ranked third in the HDR/mutation ratio at the VEGFA site.

**Fig. 3:**
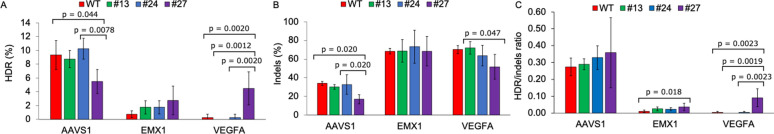
The activity of the top three obtained mutants (#13, #24, and #27 clones) compared with that of wild-type Cas9. The Cas9 gene was transfected with an episomal vector (pEBMulti. Hyg) via lipofection. Plasmid DNA coding sgRNA and template ssODN, which knock in 12 bases by removing 3 bases from the original sequence, were transfected via electroporation after 3 days of selection with hygromycin B. Editing efficiencies were analyzed via Sanger sequencing and ICE software. The red bar graphs show the results for wild-type Cas9. The green bar graphs show the results for clone #13. The blue bar graphs show the results for clone #24. The purple bar graphs show the results for clone #27. **A** The ratio of HDR-mediated target knock-in at the AAVS1, EMX1, and VEGFA target sites in HEK293 cells. **B** The ratio of target mutations at the AAVS1, EMX1, and VEGFA target sites in HEK293 cells. **C** The ratio of target knock-in per target mutation at the AAVS1, EMX1, and VEGFA target sites in HEK293 cells. All the samples except for the AAVS1 target Cas9 sample were performed in four replicates. One trial of AAVS1 targeting Cas9 could not be performed because of sample loss, but three replicates of AAVS1 targeting Cas9 were performed. The p values in the figures are the p values obtained by performing a Tukey test when the significance level was set at 5%. The raw data sets of each trial are shown in Supplementary Table 3

To confirm the HDR efficiency observed in Supplementary Fig. 5, we constructed pEB episomal vectors encoding each Cas9 mutant. We used ssODN, which introduces a 12-base insertion containing the HindIII recognition site at the AAVS1, EMX1, and VEGFA target sites. The HDR efficiency of these mutants was evaluated via interference of CRISPR edits (ICE) analysis. For this purpose, ab1 sequencing files obtained from Sanger sequencing were analyzed to determine the precise target knock-in (HDR) efficiency. Compared with the wild-type Cas9 and the other Cas9 mutants, the #27 clone presented a greater knock-in efficiency at the VEGFA target site (Fig. 3A). However, the mutation ratio induced by clone #27 tended to be lower than that induced by the other mutants (Fig. 3B). Consequently, the #27 mutant displayed the highest knock-in/mutation ratio when tested with a VEGFA-targeting sgRNA (Fig. 3C). Despite its superior HDR efficiency at the VEGFA target site, clone #27 exhibited lower activity than did wild-type Cas9 and the other mutants at the AAVS1 target site. To distinguish this Cas9 variant, we designated it HDR-Screening-Selected (HSS) Cas9. The HSS Cas9 variant has two mutations: I795V and L918E (Supplementary Fig. 4).

### HSS Cas9 showed higher HDR efficiency than did wild-type Cas9 at a particular target site

**Fig. 4:**
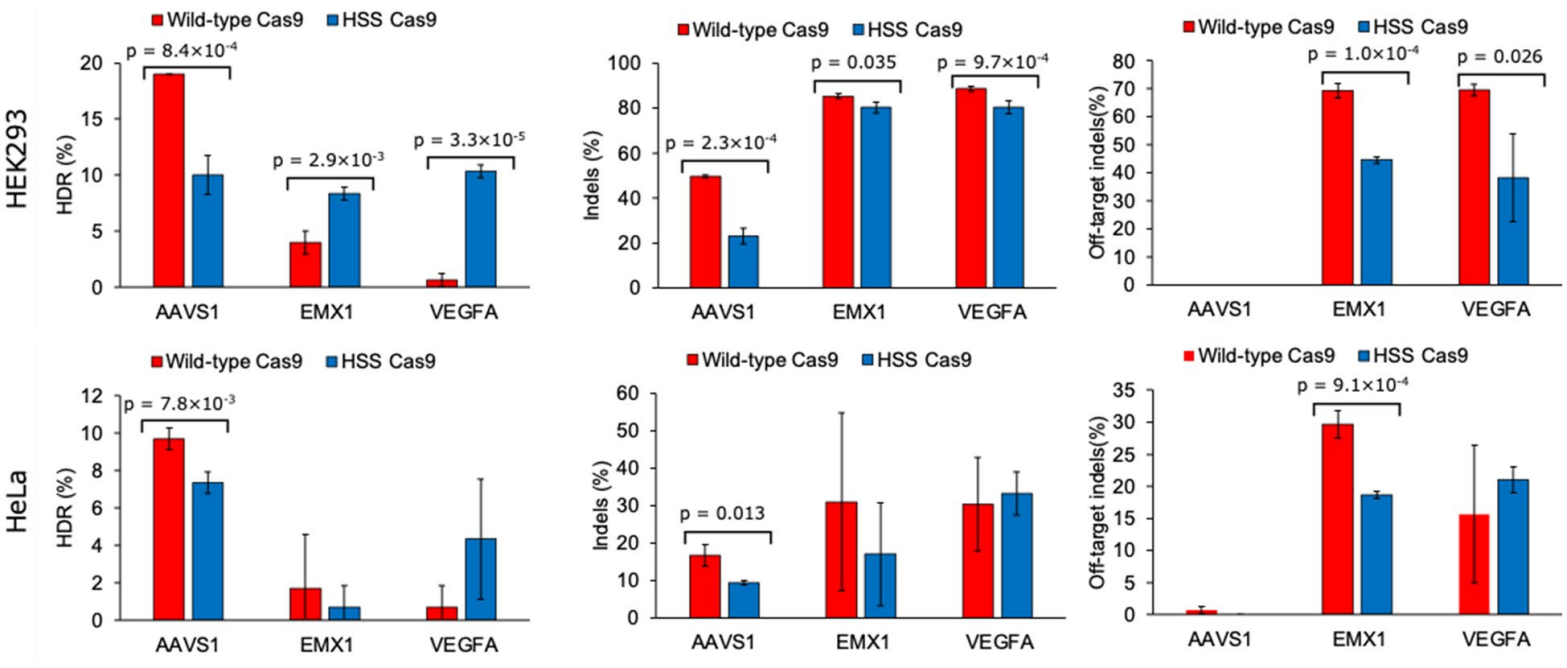
Differences in genome editing features between wild-type Cas9 and HSS Cas9 in HEK293 and HeLa cells. pEBMulti. Hyg encoding wild-type Cas9 or HSS Cas9 was transfected into the cells via lipofection. After 3 days of selection with hygromycin B, the sgRNA plasmid and ssODN were introduced via electroporation. Genome editing analysis was performed via Sanger sequencing and ICE software. All red bar graphs show data for wild-type Cas9. All blue bar graphs show HSS Cas9 data. **A** Comparison of HDR efficiency between wild-type Cas9 and HSS Cas9 when AAVS1, EMX1, and VEGFA target sgRNAs were used in HEK293 cells. **B** Comparison of on-target mutation efficiency between wild-type Cas9 and HSS Cas9 when AAVS1, EMX1, and VEGFA target sgRNAs were used in HEK293 cells. **C** Comparison of off-target mutation efficiency between wild-type Cas9 and HSS Cas9 when AAVS1, EMX1, and VEGFA target sgRNAs were used in HEK293 cells. **D** Comparison of HDR efficiency between wild-type Cas9 and HSS Cas9 when AAVS1, EMX1, and VEGFA target sgRNAs were used in HeLa cells. **E** Comparison of on-target mutation efficiency between wild-type Cas9 and HSS Cas9 when AAVS1, EMX1, and VEGFA target sgRNAs were used in HeLa cells. **F** Comparison of off-target mutation efficiency between wild-type Cas9 and HSS Cas9 when AAVS1, EMX1, and VEGFA target sgRNAs were used in HeLa cells. All the samples were analyzed in triplicate. The p values in the figures are the p values obtained via Student’s t test. The raw data sets of each trial are shown in Supplementary Table 4

To investigate the editing properties of HSS Cas9, including its off-target mutation rate, we compared its activity with that of wild-type Cas9 across three target sites: AAVS1, EMX1, and VEGFA. Genome editing was performed in HEK293A and HeLa cells via either wild-type Cas9 or HSS Cas9. HSS Cas9 exhibited lower activity than did wild-type Cas9 when targeting the AAVS1 target site in both HEK293A and HeLa cells (Fig. 4). However, when the EMX1 and VEGFA sites were edited, HSS Cas9 demonstrated higher HDR efficiency than did wild-type Cas9 in HEK293A cells (Fig. 4A). These results were consistent with the findings presented in Fig. 3. The off-target mutation rate of HSS Cas9 was lower than that of wild-type Cas9 in HEK293A cells when EMX1 and VEGFA were targeted, suggesting that, compared with the wild-type enzyme, HSS Cas9 has greater editing precision. However, differences in editing characteristics were observed between HEK293A and HeLa cells. When EMX1 was targeted in HeLa cells, HSS Cas9 displayed lower overall activity than did wild-type Cas9 (Fig. 4D, E, and F). In contrast, when VEGFA-targeting sgRNA was used, the knock-in efficiency tended to increase in HeLa cells (Fig. 4D). In contrast, the off-target mutation ratio of HSS Cas9 at the VEGFA target site in HeLa cells was greater than that of wild-type Cas9 (Fig. 4F), indicating that HSS Cas9 may behave differently depending on the cell type and chromatin environment.

To further analyze the biochemical properties of HSS Cas9, we produced recombinant wild-type and HSS Cas9 proteins (Supplementary Fig. 6). Their cleavage efficiency was assessed via in vitro cleavage assays with PCR-amplified AAVS1 and VEGFA DNA fragments as substrates. After 4 h of reaction, wild-type Cas9 cleaved approximately three times more DNA than HSS Cas9 did at both the AAVS1 and VEGFA target sites (Supplementary Fig. 7). Notably, the cleavage activity of HSS Cas9 appeared to saturate after 3 h, suggesting that compared with wild-type Cas9, HSS Cas9 may cleave DNA more slowly or remain bound to the target DNA longer after cleavage.

### ddPCR revealed that HSS Cas9 with phosphorotioated ssODN presented higher HDR efficiency than did wild-type Cas9

**Fig. 5:**
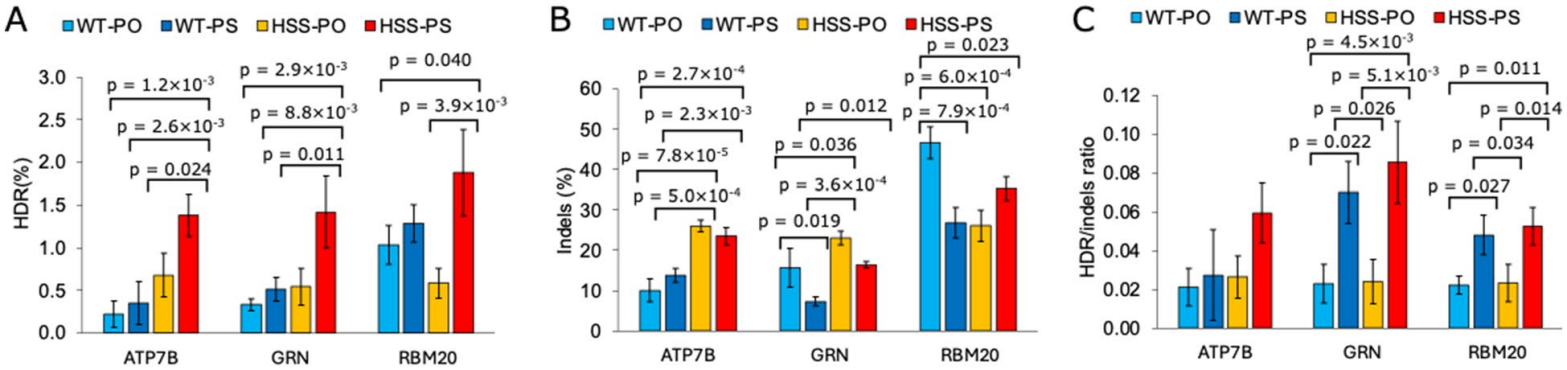
Droplet digital PCR analysis of the edited genome using wild-type Cas9 or HSS Cas9. Plasmid DNA encoding wild-type Cas9 or HSS Cas9 and each target sgRNA was transfected with ssODN into HEK293 cells via lipofection. The transfected cells were selected with puromycin for 3 days. Two types of ssODNs were used in this experiment. One is the normal phosphodiester bond oligo DNA shown as PO in the figure, and the other is the phosphorothioate bond oligo DNA at 2 bases from two ends shown as PS in the figure. WT shows wild-type Cas9. HSS shows HSS Cas9. **A** HDR efficiency of each sample when ATP7B, GRN, or RBM20 target sgRNA was used. **B** indels efficiency of each sample when ATP7B, GRN, or RBM20 target sgRNA was used. **C** HDR/indels ratio of each sample when ATP7B, GRN, or RBM20 target sgRNA was used. All the samples were analyzed in triplicate. The p values in the figures are the p values obtained by performing a Tukey test when the significance level was set at 5%. The raw data sets of each trial, including the data in Fig. 6, are shown in Supplementary Table 5. The calculated data sets of each trial, including the data in Fig. 6, are shown in Supplementary Table 6. The data in Figs. 5 and 6 were taken from the same experiments

We assessed HDR-mediated genome editing via a digital droplet PCR (ddPCR) system [18] to confirm that HSS Cas9 would also show increase HDR efficiency at different targeted genes with different analysis method. The targeted genes included ATP7B, GRN, and RBM20, which were used in a previous study [11]. We found that target sequences with relatively lower GC contents, such as EMX1 (GC content: 50%) and VEGFA (GC content: 60%), presented better HDR efficiency than did the AAVS1 target sequence (GC content: 75%) when HSS Cas9 was used (Figs. 3A and 4A, B, and C). On the basis of this observation, we selected one sgRNA that targeted a DNA sequence with approximately 60% GC content from among the sgRNAs used in reference [11]. The selected sgRNAs targeting ATP7B, GRN, or RBM20 contained GC contents of 50%, 60%, and 65%, respectively (Supplementary Table 2). To further investigate HDR efficiency, we used two types of single-stranded oligodeoxynucleotides (ssODNs). One type, referred to as PO-ssODN (shown as “-PO” in Fig. 5, hereinafter called PO-ssODN), is a common phosphodiester bond ssODN. The second type, called PS-ssODN (shown as “–PS” in Fig. 5, hereinafter called PS-ssODN), is protected from exonuclease degradation by phosphorothioate modification of two bases at both ends. All the ssODNs were designed to introduce a single base mismatch mutation near the target site [11].

When the ATP7B sgRNA was used, the HSS Cas9 sample demonstrated greater indels and HDR efficiency than did wild-type Cas9, particularly when the PS-ssODN was used (Fig. 5A and B). The HDR/indels ratio slightly increased when HSS Cas9 was used, with a 1.3-fold increase for PO-ssODN and a 2.2-fold increase for PS-ssODN. However, statistical analysis via the Tukey test revealed no significant differences between these samples (Supplementary Table 7).

HSS Cas9 also displayed higher activity than did wild-type Cas9 when the GRN was targeted with sgRNA, similar to its effect when ATP7B sgRNA was used (Fig. 5A and B). The HDR efficiency of HSS-PS was 2.7-fold greater than that of WT-PS, with a p value of 8.8 × 10⁻^3^. However, there was no significant increase in the HDR/indels ratio in “HSS-PS” compared with “WT-PS” because the indels ratio was also 2.2-fold greater than that of wild-type Cas9. On the other hand, the HDR/indels ratio was significantly greater when PS-ssODN was used than when PO-ssODN was used.

When RBM20 sgRNA was used, there was little difference in activity between wild-type Cas9 and HSS Cas9 compared with the effects observed with ATP7B and GRN sgRNA (Fig. 5A and B). “WT-PO” resulted in a 1.8-fold higher HDR efficiency than “HSS-PO”, whereas “HSS-PS” resulted in a 1.5-fold higher HDR efficiency than “WT-PS”. In terms of the HDR/indels ratio, compared with PO-ssODN, the use of PS-ssODN increased the ratio when wild-type Cas9 and HSS Cas9 were used (Fig. 5C).

### Cell cycle-dependent activation of HSS Cas9 enhances the accuracy of genome editing

**Fig. 6:**
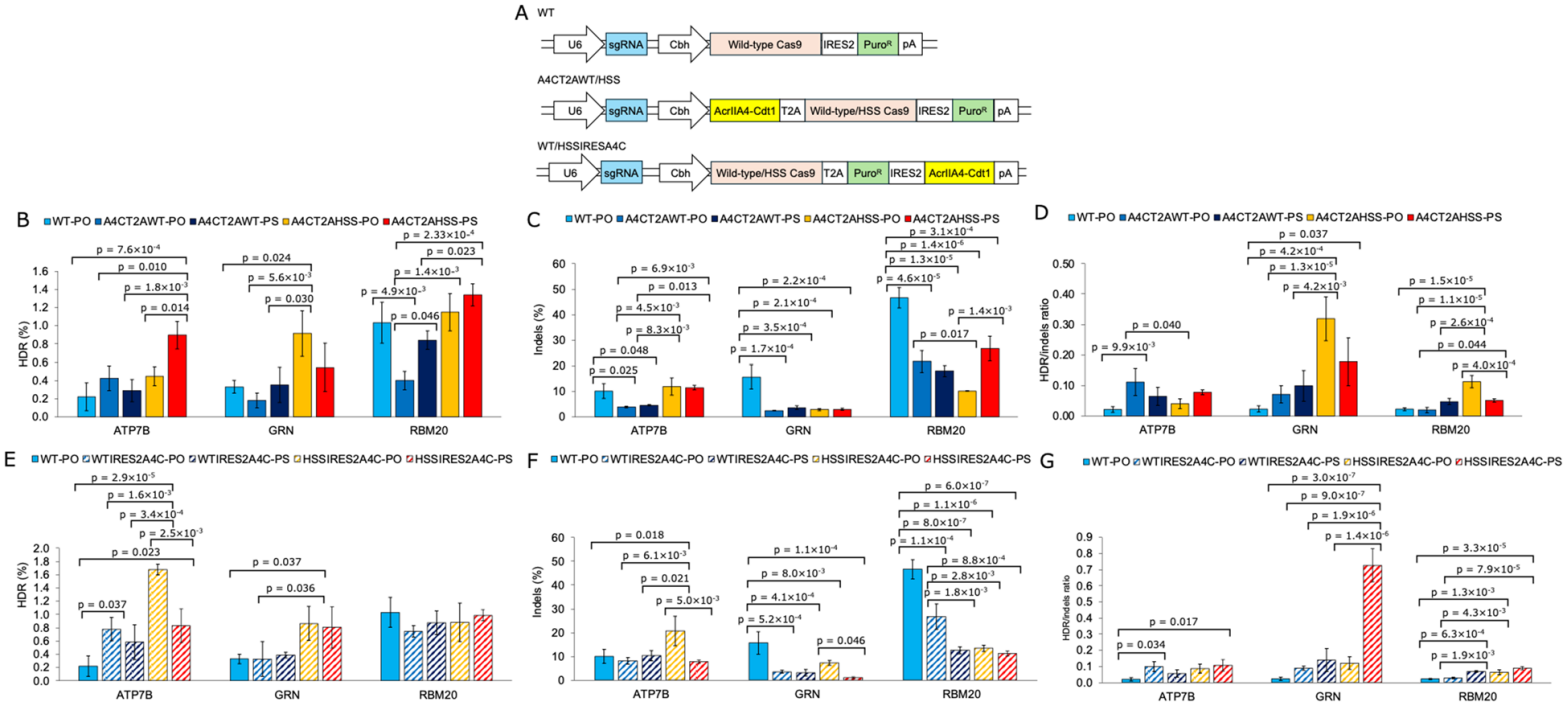
Droplet digital PCR analysis of the edited genome via cell cycle-dependent activation of wild-type Cas9 or HSS Cas9. Plasmid DNA encoding AcrIIA4-Cdt1 and wild-type Cas9 or HSS Cas9 and each target sgRNA was transfected with ssODN into HEK293 cells via lipofection. The transfected cells were selected with puromycin for 3 days. Two types of ssODNs were used in this experiment. One is the normal phosphodiester bond oligo DNA shown as PO in the figure, and the other is the phosphorothioate bond oligo DNA at 2 bases from two ends shown as PS in the figure. WT shows wild-type Cas9. HSS shows HSS Cas9. A4CT2A shows AcrIIA4-Cdt1-T2A-Cas9 (WT or HSS). IRESA4C shows Cas9 (WT or HSS)-IRES2-AcrIIA4-Cdt1. All the data in Figs. 5 and 6 were taken from the same experiments, and the WT-PO data are the same as those in Fig. 5. **A** Detailed gene cassettes of each sample. **B** HDR efficiency of each sample when ATP7B, GRN, or RBM20 target sgRNA was used with A4CT2AWT/HSS gene cassettes and PO-ssODN. **C** Indels efficiency of each sample when ATP7B, GRN, or RBM20 target sgRNA was used with A4CT2AWT/HSS gene cassettes and PO-ssODN. **D** HDR/indels ratio of each sample when ATP7B, GRN, or RBM20 target sgRNA was used with A4CT2AWT/HSS gene cassettes and PO-ssODN. **E** HDR efficiency of each sample when ATP7B, GRN, or RBM20 target sgRNA was used with WT/HSSIRESA4C gene cassettes and PS-ssODN. **F** Indels efficiency of each sample when ATP7B, GRN, or RBM20 target sgRNA was used with WT/HSSIRESA4C gene cassettes and PS-ssODN. **G** HDR/indels ratio of each sample when ATP7B, GRN, or RBM20 target sgRNA was used with WT/HSSIRESA4C gene cassettes and PS-ssODN. All the samples were analyzed in triplicate. The p values in the figures are the p values obtained by performing a Tukey test when the significance level was set at 5%. The raw data sets of each trial are shown in Supplementary Table 5. The calculated data sets of each trial, including the data in Fig. 5, are shown in Supplementary Table 6. The data in Figs. 5 and 6 were taken from the same experiments. All p-values by all two samples combinational comparison from (**B**–**G**) were shown in Supplementary Table 8 and 9. All p-values by all two samples combinational comparison in Figs. 5 and 6 were shown in Supplementary Table 10 and 11

To evaluate whether Cas9 activity can be regulated in a cell cycle-dependent manner, we used AcrIIA4-Cdt1 [9]. This system allows for cell cycle-dependent activation of Cas9 by co-expression of a protein fusing AcrIIA4, a known potent inhibitor protein of SpCas9, with the ubiquitinated domain (30–120) of Cdt1. In this study, we constructed an all-in-one plasmid that encodes the sgRNA AcrIIA4-Cdt1 and Cas9. We designed two types of dual-expression systems to coexpress AcrIIA4-Cdt1 and Cas9 (Fig. 6A). One system uses a T2A self-cleaving peptide, whereas the other system utilizes IRES2-mediated translation. For T2A peptide-based expression, we designed a gene cassette, AcrIIA4-Cdt1-T2A-Cas9 (wild-type or HSS)-IRES2-PuroR (puromycin resistance gene), which is labeled “A4CT2AWT/HSS” in Fig. 6. We also modified the gene cassette so that Cas9 (wild-type or HSS)-T2A-PuroR-IRES2-AcrIIA4-Cdt1 would allow translation of AcrIIA4-Cdt1 through the IRES2 sequence, which is labeled WT/HSSIRESA4C in Fig. 6.

We assessed the cell cycle control of the samples in Fig. 5 at the same time and split the figures for easier viewing. Therefore, all the “WT-PO” samples are identical data. All raw ddPCR data are shown in Supplementary Table 5, and the average and standard deviation of all samples are presented in Supplementary Table 6. The p values shown in Figs. 5 and 6 indicate the p value of the Tukey test by comparing the data in the same figures. Each p value is shown in Supplementary Table 7 for Fig. 5, in Supplementary Table 8 for Fig. 6B–D, and in Supplementary Table 9 for Fig. 6E–G. The p values of the Tukey test when comparing all data for HDR efficiency or the HDR/indels ratio in Figs. 5 and 6 are also shown in Supplementary Table 10 (HDR) and Supplementary Table 11 (HDR/indels). The data for T2A-mediated AcrIIA4-Cdt1 co-expression compared with only wild-type Cas9 are shown in Fig. 6B–D. The data of IRES-mediated AcrIIA4-Cdt1 co-expression compared with only wild-type Cas9 are shown in Fig. 6E‒G.

When the ATP7B sgRNA was used, indels efficiency tended to decrease with the co-expression of AcrIIA4-Cdt1 with wild-type or HSS Cas9 (Fig. 6C and F). In contrast, HDR efficiency increased by more than fourfold in “A4CT2AHSS-PS” compared with “WT-PO” (Fig. 6B). In the case of IRES-mediated co-expression of AcrIIA4-Cdt1, “WTIRESA4C-PO”, “HSSIRESA4C-PO”, and “HSSIRESA4C-PS” resulted in a significant increase in HDR efficiency compared with that of “WT-PO” (Fig. 6E). In particular, the HDR efficiency of “HSSIRESA4C-PO” was 7.5-fold greater than that of “WT-PO”. The three most precisely edited samples were “A4CT2AWT-PO”, “HSSIRESA4C-PS”, and “WTIRESA4C-PO”, all of which presented HDR/indels ratios greater than 4.7-fold greater than those of the “WT-PO” sample (Fig. 6D and G).

When GRN-targeted sgRNA was used, indels efficiency was significantly reduced by the co-expression of AcrIIA4-Cdt1 (Fig. 6C and F). Compared with “WT-PO”, “A4CT2AHSS-PO” and “HSSIRESA4C-PO” presented more than 2.5-fold HDR efficiency (Fig. 6B and E). In terms of the HDR/indels ratio, the three samples with the highest HDR/indels ratios were “A4CT2AHSS-PO”, “A4CT2AHSS-PS”, and “HSSIRESA4C-PS” (Fig. 6D and G). Notably, the “HSSIRESA4C-PS” sample presented an HDR/indels ratio 31.8-fold greater than that of the “WT-PO” sample.

 When RBM20 sgRNA was used, indels efficiency was significantly reduced by the co-expression of AcrIIA4-Cdt1 as a GRN sgRNA (Fig. 6C and F). No cell cycle control samples presented a significant increase in HDR efficiency compared with that of “WT-PO”. On the other hand, HSS Cas9 with cell cycle control presented greater HDR efficiency than did wild-type Cas9 with cell cycle control when AcrIIA4-Cdt1 was expressed with Cas9 through the T2A peptide (Fig. 6B). However, this tendency was not observed when AcrIIA4-Cdt1 was expressed with Cas9 through IRES2 (Fig. 6E). The three most precisely edited samples were “A4CT2AHSS-PO,” “HSSIRESA4C-PS,” and “WTIRESA4C-PS,” each of which presented HDR/indels ratios greater than 3.1-fold greater than those of the “WT-PO” sample (Fig. 6D and G).

### Optimization of cell cycle-dependent activation system enhances the accuracy of genome editing by HSS Cas9

**Fig. 7:**
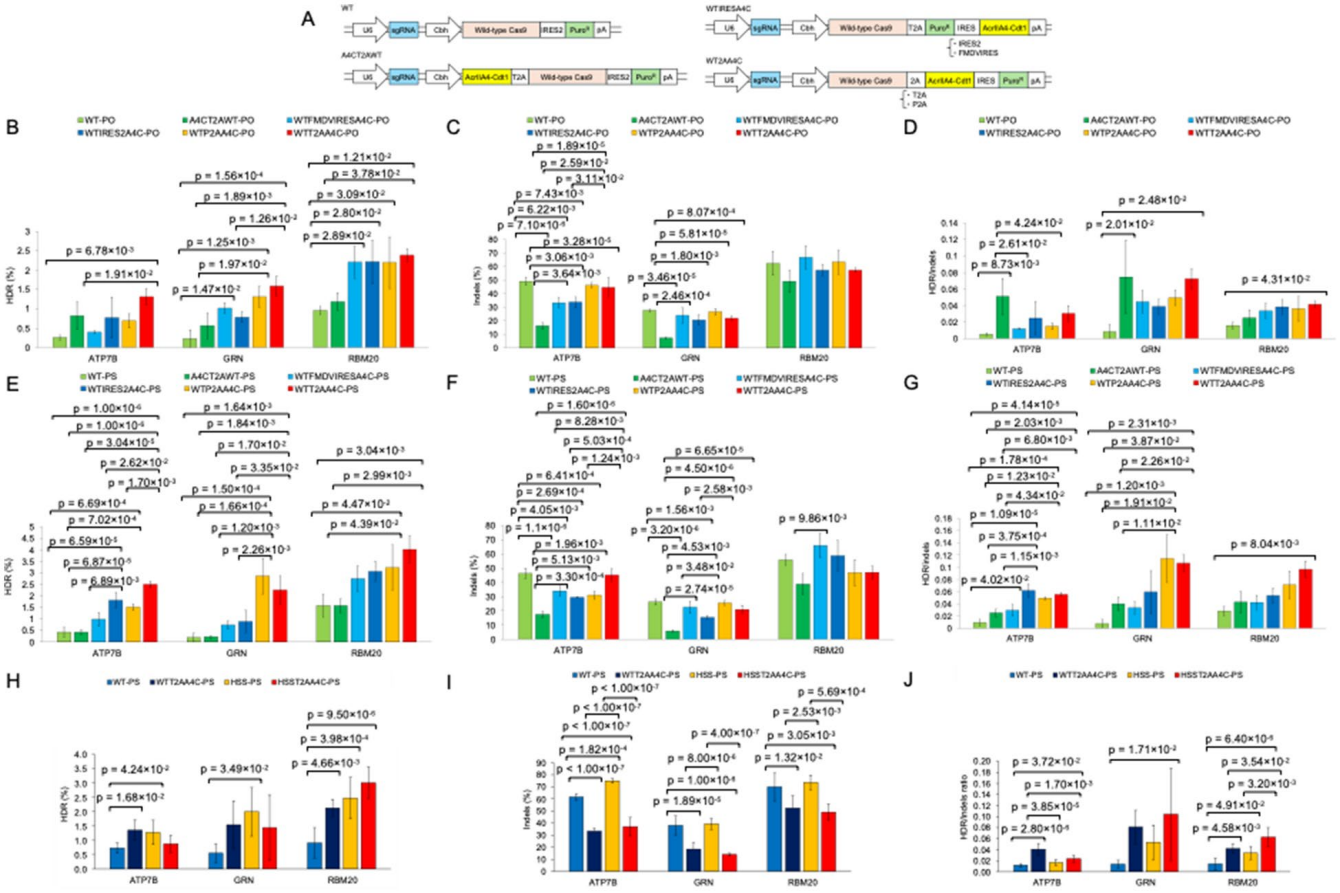
Optimization of the gene cassette for cell cycle-dependent activation in the co-delivery of Cas9, sgRNA, and ssODN. Plasmid DNA encoding AcrIIA4-Cdt1 and either wild-type Cas9 or HSS Cas9 along with each target sgRNA was transfected into HEK293 cells together with ssODN via lipofection. Transfected cells were selected with puromycin for 3 days. Two types of ssODNs were used: a standard phosphodiester oligo (denoted as PO in the figure), and an oligo with phosphorothioate bonds at two terminal bases (PS). WT refers to wild-type Cas9. HSS refers to HSS Cas9. A4CT2A indicates AcrIIA4-Cdt1-T2A-Cas9 (WT or HSS). IRESA4C indicates Cas9 (WT or HSS)-IRES2-AcrIIA4-Cdt1. All data shown in (**B**–**G**) were obtained from the same experimental set, as were those in (**H**–**J**). **A** Schematic of gene cassettes used in each sample. **B**–**D** HDR efficiency, indel frequency, and HDR/indel ratio, respectively, for each sample when ATP7B, GRN, or RBM20 sgRNAs were used with WT Cas9 and PO-ssODN across five types of gene cassettes. **E**–**G** Same as B–D, but with PS-ssODN. All data in (**B**–**G**) were analyzed in triplicate. **H**–**J** HDR efficiency, indel frequency, and HDR/indel ratio, respectively, for each sample using WT or HSS A4CT2A gene cassettes and PS-ssODN. n = 6. p-values shown in the figures were calculated using Tukey’s test with a significance level of 5%. Raw data for each trial are provided in Supplementary Table 12, and the corresponding processed data in Supplementary Table 13. All pairwise comparisons and their p-values from Fig. (**B**–**G**) and (**H**–**J**) are summarized in Supplementary Tables 14, 15, and 16

Co-expression of AcrIIA4-Cdt1 with Cas9 showed an increase of HDR efficiency even when a plasmid encodes Cas9, AcrIIA4-Cdt1 and sgRNA was delivered with ssODN (Fig. 6B and E). However, the increased amount of HDR was not large, especially in comparison between wild-type Cas9 and A4CT2AWT, which was previously reported [9]. We used an episomal vector in the previous research, and selected the transfected cells by hygromycin B for 4–5 days before introduction of the sgRNA encoding plasmid and ssODN by electroporation. Because several cell cycles likely occurred during hygromycin B selection, continuous expression of Cas9 and the expression and subsequent degradation of AcrIIA4-Cdt1 would have taken place. As a result, by the time the sgRNA-encoding plasmid and ssODN were introduced, the ratio of AcrIIA4-Cdt1 to Cas9 expression may have been relatively optimal. This suggests that the simultaneous delivery of a plasmid encoding AcrIIA4-Cdt1, Cas9, and sgRNA together with an ssODN may have been less effective in inducing HDR compared to the previously reported method. Moreover, WTIRES2A4C exhibited higher HDR efficiency than A4CT2AWT when a PO-ssODN was used as the template (Fig. 6B and E). These results suggest that a gene cassette favoring higher Cas9 expression than AcrIIA4-Cdt1 may lead to improved HDR efficiency. Therefore, we optimized the gene cassette for cell cycle-dependent activation to enable the simultaneous delivery of a plasmid encoding AcrIIA4-Cdt1, wild-type Cas9, and sgRNA together with ssODN. We newly constructed three more types of plasmid DNA which encodes WTFMDVIRESA4C, WTP2AA4C, and WTT2AA4C (Fig. 7A). WTT2AA4C showed the highest HDR ratio at ATP7B, GRN, and RBM20 target sites in both cases when PO- or PS-ssODN was used as template, except for the GRN target site with PS-ssODN (Fig. 7B and E). HDR efficiency of “WTT2AA4C” at each target site increased more than 5.1-fold (ATP7B), 6.7-fold (GRN), or 2.5-fold (RBM20) than “WT”. On the other hand, the indels ratio of “WTT2AA4C” was not largely different from that of “WT” (Fig. 7C and F). As a result, the HDR/indels ratio of “WTT2AA4C” was also increased at around the same fold as HDR efficiency compared with “WT” (Fig. 7D and G).

Next, the Cas9T2AA4C gene cassette was applied to HSS Cas9. The effect of gene cassette optimization on the increase of editing accuracy was performed using PS-ssODN. Similar to the results in Fig. 5, HSS Cas9 exhibited higher HDR efficiency than wild-type Cas9 (Fig. 7H). However, when the activity of HSS Cas9 was regulated in a cell cycle-dependent manner, as shown in Fig. 6, the overall HDR efficiency decreased, particularly when targeting ATP7B and GRN (Fig. 7H). In addition, this cell cycle-dependent activation led to about 50% reduction in the indel frequency of HSS Cas9 at these target sites (Fig. 7I). As a result, the ratio of HDR efficiency to indel frequency significantly improved under cell cycle-dependent activation of HSSCas9 compared with wild-type Cas9 alone sample (Fig. 7J).

## Discussion

Researchers have developed various Cas9 variants, particularly SpCas9, to achieve precise gene editing through molecular design and directed evolution [21]. Most molecularly designed Cas9 variants aim to reduce off-target cleavage by minimizing the interaction between SpCas9 and DNA [22–25]. Recently, more diverse variants have been identified through screening randomized mutant libraries [26–31], which are primarily designed to mitigate off-target effects. Notably, most of these screening systems have been conducted in *E. coli* or yeast. In this study, we constructed a screening system in human cells to obtain Cas9 variants with improved HDR efficiency while minimizing off-target editing (Fig. 1). Through this system, we identified a novel Cas9 variant containing unreported mutations in the nuclease domain that exhibited higher HDR efficiency than did wild-type Cas9 at specific target sites. After screening, we obtained several original Cas9 sequences. However, further optimization is necessary to construct a more efficient Cas9 mutant library on the basis of the length of the target gene. The use of alternative polymerases for error-prone PCR, such as Genemorph II (Agilent), could be an option to introduce a broader range of mutations in the target gene. In this study, we focused on introducing mutations within the nuclease domain because our previous data indicated that mutations in the RuvCIII subdomain might increase HDR efficiency at certain target sites [10]. Additionally, our screening system in this study was limited by the library size and the number of cells we could electroporate using our current instrumentation. In another study, we reported that HypaCas9 exhibited greater HDR efficiency than did wild-type Cas9 at some target sites [11]. Therefore, expanding the mutagenesis region to include both the nuclease and recognition domains might be necessary for constructing a more HDR-inducible Cas9 library. Another limitation of our screening system is that we could not remove mutants that contained silent mutations and obtained Cas9 mutants that could not induce HDR. We solve the problem of slilent mutation by changing the error-prone PCR conditions to increase the mutation ratio. In the case of mutants whose HDR activity is almost the same as that of wild-type Cas9, repeated screening from a large library via this system would solve this problem because we only tried screening once to obtain a novel mutant in this study. We also note that optimization of the transfection conditions is necessary when using other electroporators such as the Nucleofector (Lonza) or MaxCyte (MaxCyte).

Through our screening, we identified a novel mutant, HSS Cas9, which harbors two previously unreported mutations (I795V/K918E). We hypothesize that the K918E mutation significantly affects the genome editing characteristics of HSS Cas9 through reversal of the amino acid's net charge. Additionally, structural analysis suggests that I795 is located on the surface of SpCas9 on the basis of the available crystal structures of the SpCas9-sgRNA-DNA complex (PDB 4UN3 (X-ray diffraction) [32], 5F9R (X-ray diffraction) [33], 5Y36 (cryo-EM) [34], and 7OXA (X-ray diffraction) [35]. In the case of K918, several structural studies suggest potential interactions with nearby amino acids containing carboxyl groups, such as aspartic acid and glutamic acid. However, the specific interacting amino acids vary between different crystal structures. A common observation among these structures is that K918 is positioned relatively close to H840 (Supplementary Fig. 8, PDB 4UN3), an essential residue for cleaving the target DNA strand [1, 36]. Recently, an HDR-inducible SpCas9 variant, vCas9, was engineered by introducing four mutations (S55R-R976A-K1003A-T1314R) to alter the cleavage pattern and generate sticky DNA ends [37]. The K918E mutation, which alters the local electrostatic environment, may similarly influence the structure of the SpCas9-sgRNA-DNA complex, affecting its cutting profile and DNA binding affinity. Our in vitro cleavage assay further supported the idea that, compared with wild-type Cas9, HSS Cas9 has distinct reaction kinetics (Supplementary Fig. 7). Compared with HSS Cas9, wild-type Cas9 cleaved PCR products more efficiently, and both variants cleaved VEGFA PCR products more efficiently than did the AAVS1 PCR products. The AAVS1 target site has a GC content of 75%, whereas the VEGFA target site has a GC content of 60% (Supplementary Table 2). This suggests that stronger DNA‒RNA hybridization in the AAVS1 sequence may result in slower Cas9 dissociation after cleavage. We also analyzed the cell cycle phases of the cells that were transfected empty vector, wild-type Cas9 or HSS Cas9. Wild-type Cas9-expressing cells showed a lower proportion of cells in the G1 phase compared to non-transfected cells and cells transfected with an empty vector (Supplementary Fig. 9 and Supplementary Table 17). This difference was moderated as the culture period was extended. Although there was no statistically significant difference between wild-type Cas9 and HSS Cas9, a trend toward a reduced G1 population was observed in cells 40 h post-transfection. In contrast, a tendency for an increased proportion of cells in the G2/M phase was noted in wild-type Cas9-expressing cells compared to those expressing HSS Cas9, although this difference was also not statistically significant. At 64 h post-transfection, no substantial differences in cell cycle distribution were observed between wild-type Cas9 and HSS Cas9. A possible explanation for the higher proportion of cells in the G2/M phase observed with wild-type Cas9 is Cas9-induced DNA damage occurs more frequently than HSS Cas9 during the G2 phase. We hypothesize that HSS Cas9 has a slower reaction than does wild-type Cas9, potentially due to a reduced turnover rate or slower double-strand DNA cleavage, as the K918E mutation may influence the electrostatic environment of the HNH domain.

HSS Cas9 demonstrated more precise genome editing than did wild-type Cas9 at specific target sites (Figs. 3, 4, and 5). When the AAVS1 site with a 75% GC content was targeted, wild-type Cas9 exhibited higher HDR efficiency than HSS Cas9 did (Figs. 3A and 4A, and D). A similar trend was observed at the RBM20 target site, which has 65% GC content (Fig. 5A, “WT-PO” and “HSS-PO”). Conversely, HSS Cas9 exhibited a relatively high HDR efficiency at target sites with relatively low GC contents, such as EMX1 and ATP7B. We previously reported similar results when high-fidelity Cas9 variants were used [10]. Further investigation is needed to understand the relationship between Cas9 kinetics and its genome-editing properties.

Additionally, we developed a plasmid-based system for cell cycle-dependent Cas9 activation. We compared two expression strategies: co-expression of Cas9 and AcrIIA4-Cdt1 via a T2A self-cleaving peptide, as previously applied in our episomal vector system [9], and IRES2-mediated translation of AcrIIA4-Cdt1. Both systems effectively increased the HDR/indels ratio by reducing indels (Figs. 6 and 7). Furthermore, optimization of the gene cassette indicated that placing the Cas9 gene upstream of the AcrIIA4 gene could contribute more effectively to HDR enhancement (Fig. 7B, E). However, neither system significantly increased HDR efficiency compared with the episomal vector system [8–10]. In our previous studies in which episomal vectors were used, we selected cells with hygromycin for three days before the introduction of the ssODN and sgRNA. As a result, the AcrIIA4-Cdt1 protein level may have decreased during selection. This could explain why the episomal vector system was more effective in enhancing HDR. In the plasmid system, all the components, including ssODN, were transfected simultaneously. Consequently, there was a minimal difference in expression timing between AcrIIA4-Cdt1 and Cas9, as Cas9 is a large protein that requires more time for proper folding after translation. This may have limited the HDR enhancement observed with cell cycle regulation. This delay in Cas9 activation also makes PS-ssODN more suitable, as it is more stable than PO-ssODN [38]. Further optimization is required to achieve one-pot accurate editing using ssODN and plasmid DNA encoding Cas9 and AcrIIA4-Cdt1 by fine-tuning the inhibitory effect of AcrIIA4 on SpCas9.

More than a decade has passed since SpCas9 was first used in eukaryotic cells. Numerous studies have reported Cas9 variants derived from various bacterial and archaeal species, as well as alternative CRISPR systems. However, differences between bacterial and eukaryotic systems have posed challenges. For example, humans have preexisting immunity against SpCas9 [39–41], and nucleosome occupancy in the eukaryotic genome can hinder Cas9 access to target DNA [42–44]. Additionally, some studies suggest that posttranslational modifications of Cas9 and Cas12a can influence specificity and activity [45, 46]. Therefore, we believe that evolving CRISPR systems directly in human cells is crucial for optimizing genome editing for therapeutic applications. We aimed to further explore the relationship between SpCas9 mutation and HDR efficiency at various target sites by identifying additional mutants via our screening system.

## Conclusion

In this study, we constructed a system to screen Cas9 mutants that can increase HDR efficiency. In the selected Cas9, several target sequences presented higher HDR efficiency than did wild-type Cas9. Furthermore, we were able to improve the accuracy of editing further by combining the system with a cell cycle-dependent activation system. On the other hand, the HDR efficiency of wild-type Cas9 was greater than that of HSS Cas9 for some target sequences, suggesting that the relationship between DNA cleavage by Cas9 and repair by HDR may be clarified by investigating the underlying causes of this phenomenon. In addition, it is expected that more libraries can be screened by using higher-throughput methods such as AAV for the introduction of templates and sgRNAs.

## Supplementary Information

Supplementary Material 1.

Supplementary Material 2.

Supplementary Material 3

Supplementary Material 4.

Supplementary Material 5.

Supplementary Material 6.

Supplementary Material 7.

Supplementary Material 8.

Supplementary Material 9.

Supplementary Material 10.

## Data Availability

The data sets and materials used and/or analyzed during the current study are available in the supplementary information and from the corresponding author upon reasonable request.

## Abbreviations

DT: Diphteria toxin
HDR: Homology-directed repair
HSS Cas9: HDR screening selected Cas9
IRES: Internal ribosome entry site
NHEJ: Nonhomologous end joining
Indels: Insertions and deletions
PO: Phosphodiester
PS: Phosphorothioate
sgRNA: Single guide RNA
WT: Wild-type
