# Supplementary material for: Screening strategy to identify Cas9 variants with higher HDR activity based on diphtheria toxin
Source: J Biomed Sci. 2025 Dec 3;32:102. doi: 10.1186/s12929-025-01197-9 (PMC12673799; doi:10.1186/s12929-025-01197-9)
Supplement: Supplementary file 3 — Supplementary Material 3 [file 12929_2025_1197_MOESM3_ESM.pdf]

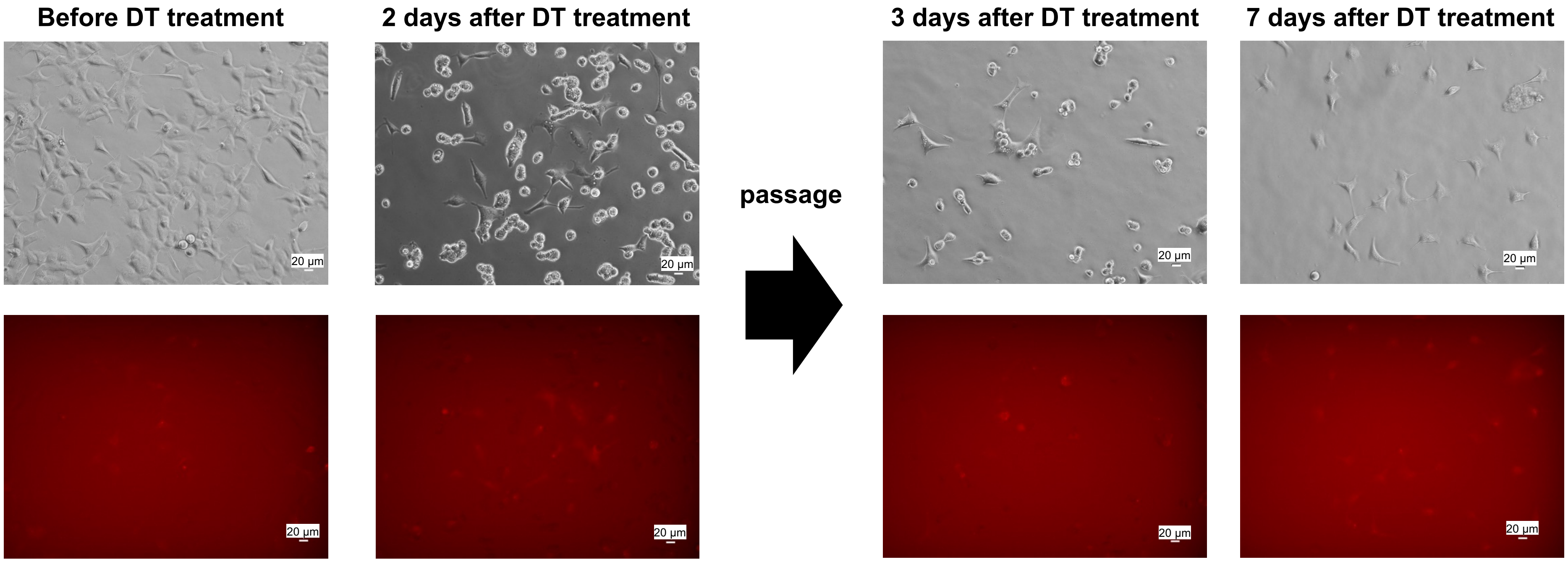

Supplementary Figure 1. Confirmation of condensation of HDR-mediated edited cell by Diphtheria toxin (DT) treatment. 20 ng/mL DT was added 3 days after the introduction of hHBEGF target sgRNA and template DNA.
