# Supplementary material for: Screening strategy to identify Cas9 variants with higher HDR activity based on diphtheria toxin
Source: J Biomed Sci. 2025 Dec 3;32:102. doi: 10.1186/s12929-025-01197-9 (PMC12673799; doi:10.1186/s12929-025-01197-9)
Supplement: Supplementary file 6 — Supplementary Material 6. [file 12929_2025_1197_MOESM6_ESM.pdf]

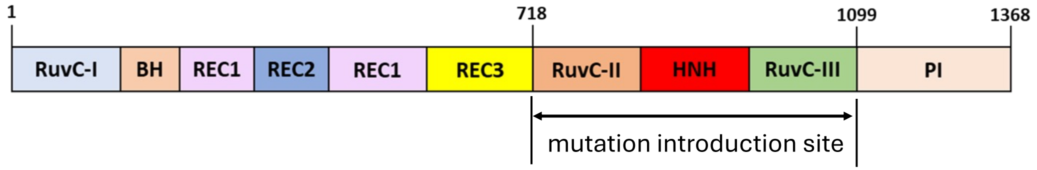

Colony #	DNA mutation	Amino acid change (nonsense mutations are not listed)
1	3rd codon C to T in 937S, 3rd codon C to A in 1038F	F1038L
2-4	3rd codon C to T in 937S, 2nd codon A to G in 973Y, 3rd codon C to A in 1038F	Y973C/F1038L
5	3rd codon G to T in 993V, 3rd codon C to T in 1067G	only nonsense
6-9	2nd codon T to C in 724I, 3rd codon A to G in 785E, 3rd codon T to C in 837D, 3rd codon C to T in 975R, 2nd codon A to G in 1075D	I724T/D1075G
10	1st codon G to A in 760V, 2nd codon A to G in 829D, 1st codon A to T in 867S, 3rd codon C to T in 995T	V760M/D829G/S867C
11	3rd codon A to G in 1068D	only nonsense
12	2nd codon T to C in 847L, 1st codon A to G in 941T, 2nd codon T to C in 958L, 3rd codon T to C in 1045F, 3rd codon A to T in 1065T	L847S/T941A/L958P
13	3rd codon C to G in 916F, 2nd codon A to G in 954K, 3rd codon C to T in 985H	F916L/K954R
14	3rd codon T to A in 840H, 1st codon G to T in 931V	H840Q/V931L
15	2nd codon A to T in 982H	H982L
16	2nd codon T to C in 911L, 2nd codon A to C in 985H, 1st codon A to T in 1063I, 3rd codon A to T in 1065T	L911S/H985P/I1063F
17	2nd codon T to A in 813L, 3rd codon G to A in 994V, 2nd codon T to C in 1021M, 2nd codon T to C in 1050I	L813P/M1021T/I1050T
18	2nd codon T to A in 857L	L857Q
19-21	not read by Sanger sequence	×
22	2nd codon T to G in 849L	L849R
23	2nd codon G to T in 777S, 2nd codon T to C in 813L, 2nd codon G to A in 915G, 2nd codon A to C in 974K	S777I/L813P/G915D/K974T
24	2nd codon in 714V, 1st codon A to T in 740T, 3rd codon G to A in 896K , 3rd codon C to T in 988Y, 2nd codon A to G in 1075D	V714A/T740S/D1075S
25	not read by Sanger sequence	×
26	original	original
27	1st codon A to G in 795I, 1st codon A to G in 918K	I795V/K918E
28	not read by Sanger sequence	×
29	original	original
30	3rd codon A to G in 895R	only nonsense

Supplementary Figure 4. The region of SpCas9 where mutations were introduced, and the mutation sites in each cloned Cas9 mutant. HSS Cas9 is clone #27.
