# Supplementary material for: Screening strategy to identify Cas9 variants with higher HDR activity based on diphtheria toxin
Source: J Biomed Sci. 2025 Dec 3;32:102. doi: 10.1186/s12929-025-01197-9 (PMC12673799; doi:10.1186/s12929-025-01197-9)
Supplement: Supplementary file 7 — Supplementary Material 7. [file 12929_2025_1197_MOESM7_ESM.pdf]

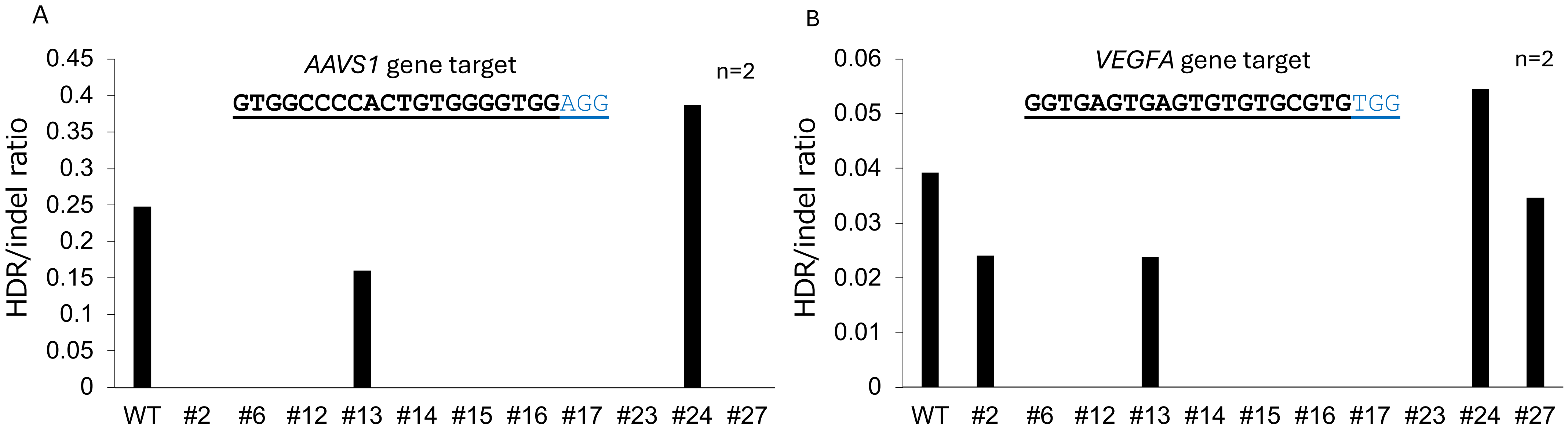

Supplementary Figure 5. Activity check of obtained Cas9 mutants. hTERT-RPE1 cells were transfected each Cas9 mutant gene by lentivirus infection. AAVS1 (A) or VEGFA (B) encoding plasmid DNA and ssODN were transfected by electroporation. HDR ratio was assessed by HindIII digestion whose recognition site would be inserted into target site by HDR. The mutation ratio was assessed by the Tide software.
