# Supplementary material for: Screening strategy to identify Cas9 variants with higher HDR activity based on diphtheria toxin
Source: J Biomed Sci. 2025 Dec 3;32:102. doi: 10.1186/s12929-025-01197-9 (PMC12673799; doi:10.1186/s12929-025-01197-9)
Supplement: Supplementary file 10 — Supplementary Material 10. [file 12929_2025_1197_MOESM10_ESM.pdf]

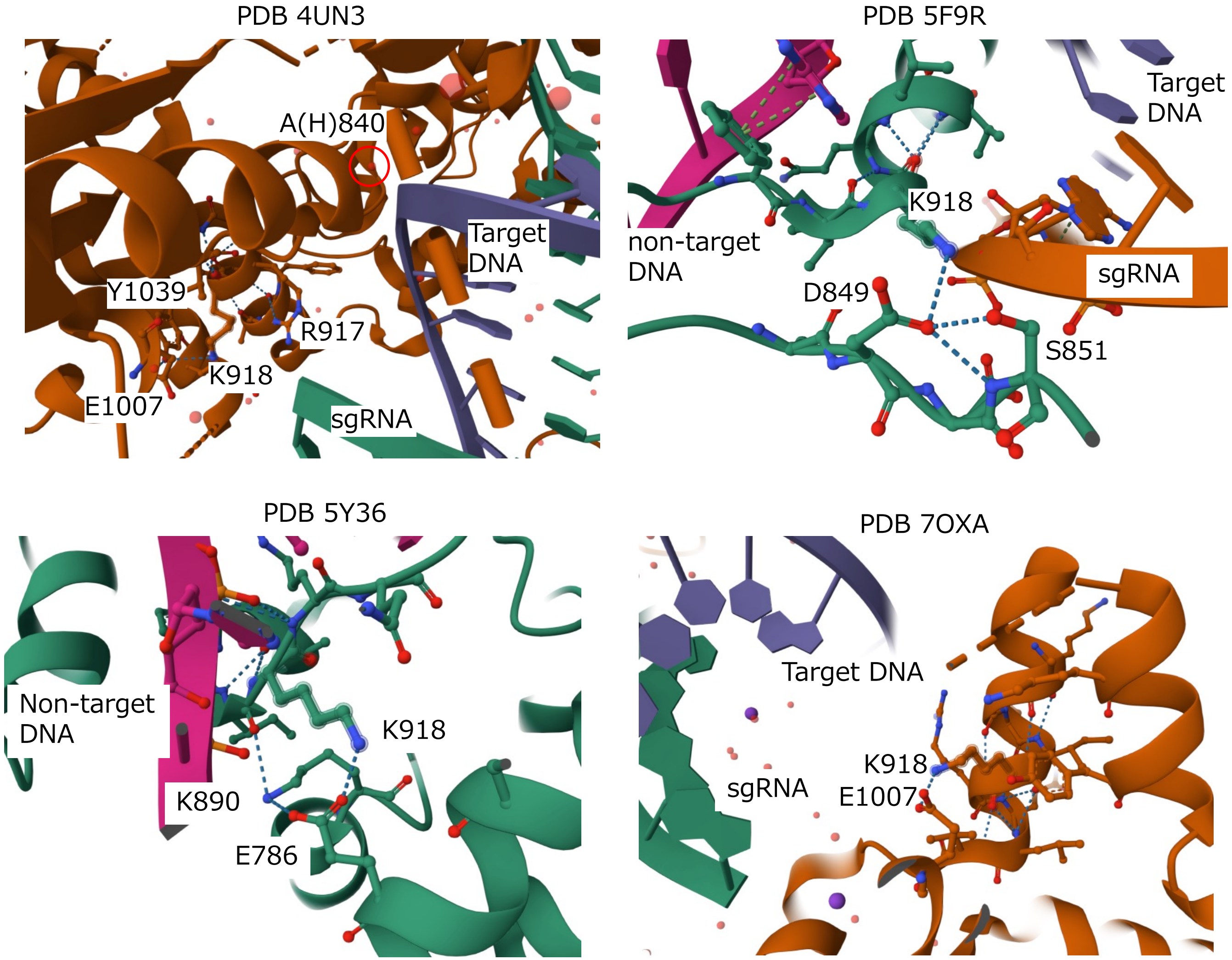

Supplementary Figure 8. Crystal structure of SpCas9-sgRNA-target DNA complexes. Each figure is modified from PDB 4UN3, 5F9R, 5Y36, and 7OXA.
