# Supplementary material for: Screening strategy to identify Cas9 variants with higher HDR activity based on diphtheria toxin
Source: J Biomed Sci. 2025 Dec 3;32:102. doi: 10.1186/s12929-025-01197-9 (PMC12673799; doi:10.1186/s12929-025-01197-9)
Supplement: Supplementary file 7 [file 12929_2025_1197_MOESM1_ESM.pdf]

Supplementary Figure 1. Confirmation of condensation of HDR-mediated edited cell by Diphtheria toxin (DT) treatment. 20 ng/mL DT was added 3 days after the introduction of hHBEGF target sgRNA and template DNA.

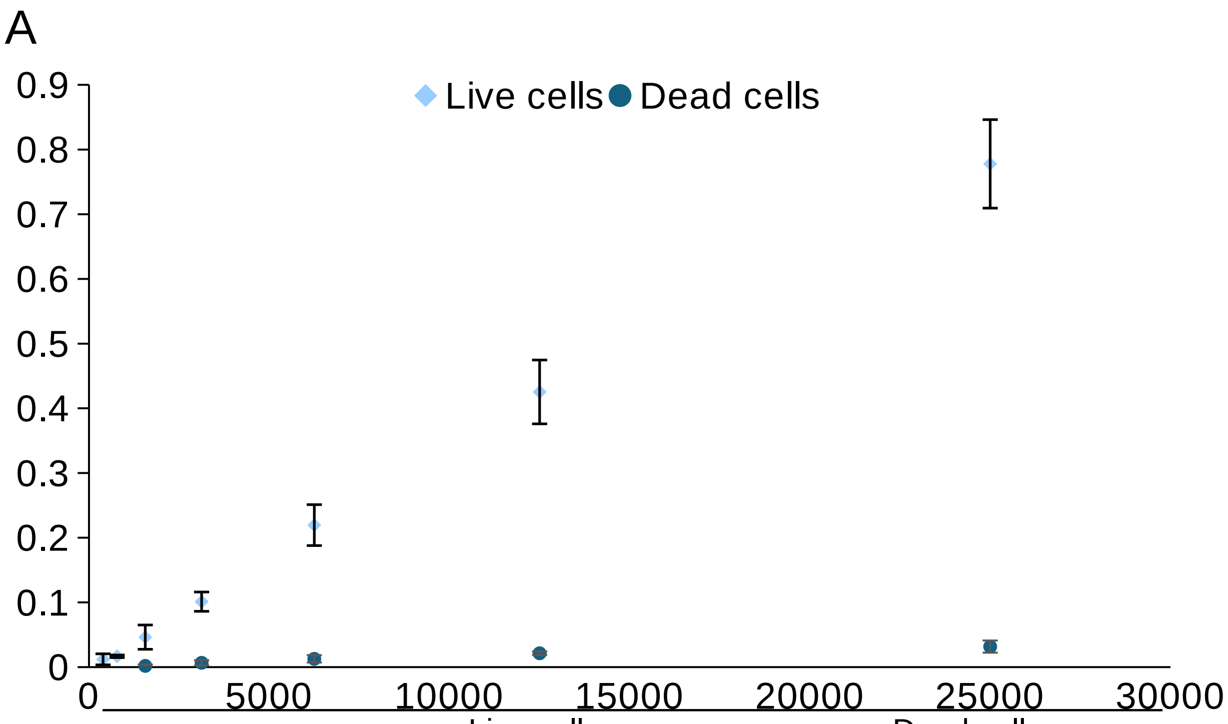

	Live cells		Dead cells	
Seed cell number	Ave.	S.D.	Ave.	S.D.
25000	0.778	0.0684	0.0316	0.00940
12500	0.425	0.0494	0.0213	0.00273
6250	0.219	0.0316	0.0127	0.00566
3125	0.101	0.0149	0.00661	0.00405
1563	0.0462	0.0188	0.00186	0.00211
781	0.0166	0.00199	-0.000146	0.00331
391	0.0120	0.00854	-0.000581	0.00722

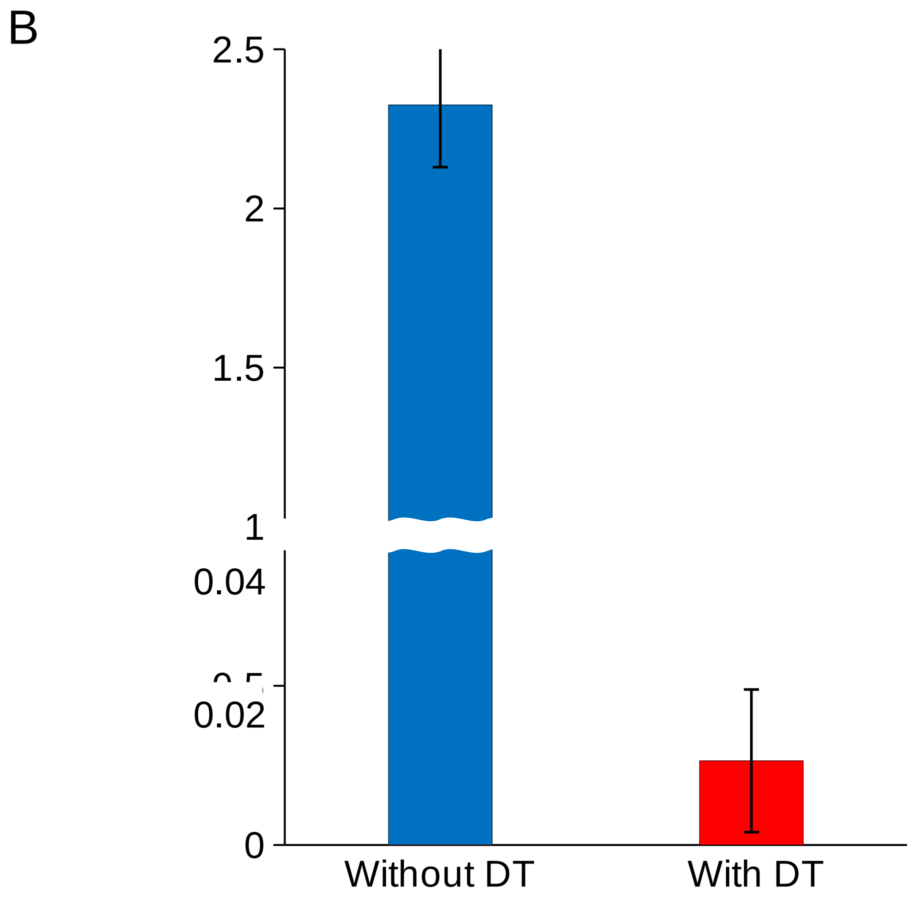

sample	Ave.	S.D.
Without DT	2.32	0.0127
With DT	0.195	0.0108

Supplementary Figure 2. Validation of selective elimination of non-edited cells by diphtheria toxin (DT) treatment. (A) Correlation between the number of hTERT-RPE1 cells and absorbance at 450 nm of WST-8 formazan. Dead cells were prepared using the supplied lysis buffer. (B) Confirmation of cell death following treatment with 20 ng/mL DT. hTERT-RPE1 cells (4,000 cells/well) were seeded, and DT was added the following day. After 3 days of DT treatment, cell viability was assessed. Data represent the mean of three replicates.

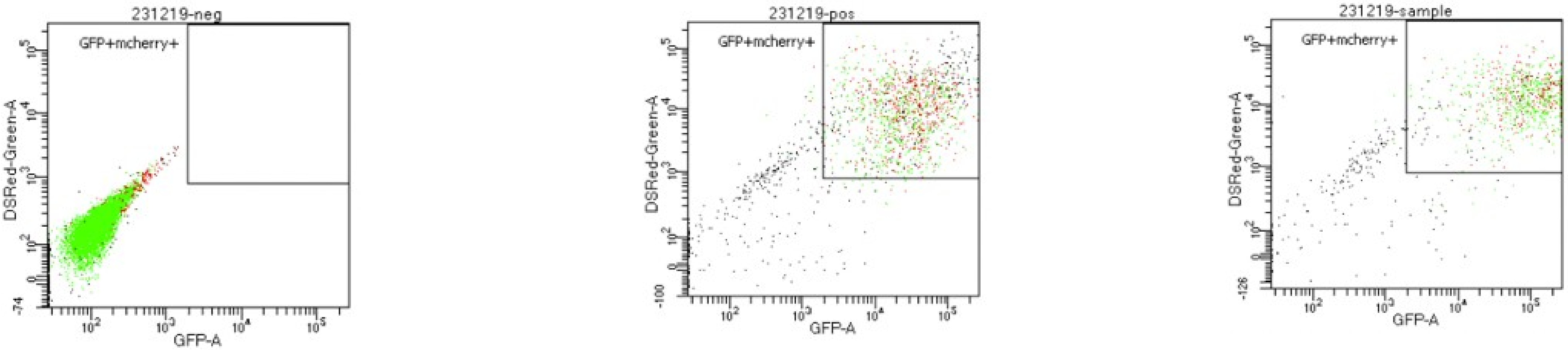

Tube: neg

Population	#Events	%Parent	%Total
All Events	10,000	####	100.0
P1	9,633	96.3	96.3
P2	8,992	93.3	89.9
P3	8,701	96.8	87.0
GFP+mcherry+	0	0.0	0.0

Tube: pos

Population	#Events	%Parent	%Total
All Events	1,513	####	100.0
P1	989	65.4	65.4
P2	534	54.0	35.3
P3	474	88.8	31.3
GFP+mcherry+	452	95.4	29.9

Tube: sample

Population	#Events	%Parent	%Total
All Events	1,006	####	100.0
P1	763	75.8	75.8
P2	490	64.2	48.7
P3	410	83.7	40.8
GFP+mcherry+	403	98.3	40.1

Supplementary Figure 3. Results of FACS sorting of mutant library. The “neg” shows data of hTERT-RPE1 cells as a negative control. The “pos” shows data of edited hTERT-RPE1 cells with wild-type Cas9 as positive control. The “sample” shows actual data of the corrected cells by screening from random Cas9 mutant library.

Supplementary Figure 4. The region of SpCas9 where mutations were introduced, and the mutation sites in each cloned Cas9 mutant. HSS Cas9 is clone #27.

HDR/indel ratio

HDR/indel ratio

Supplementary Figure 5. Activity check of obtained Cas9 mutants. hTERT-RPE1 cells were transfected each Cas9 mutant gene by lentivirus infection. AAVS1 (A) or VEGFA (B) encoding plasmid DNA and ssODN were transfected by electroporation. HDR ratio was assessed by HindIII digestion whose recognition site would be inserted into target site by HDR. The mutation ratio was assessed by the Tide software.

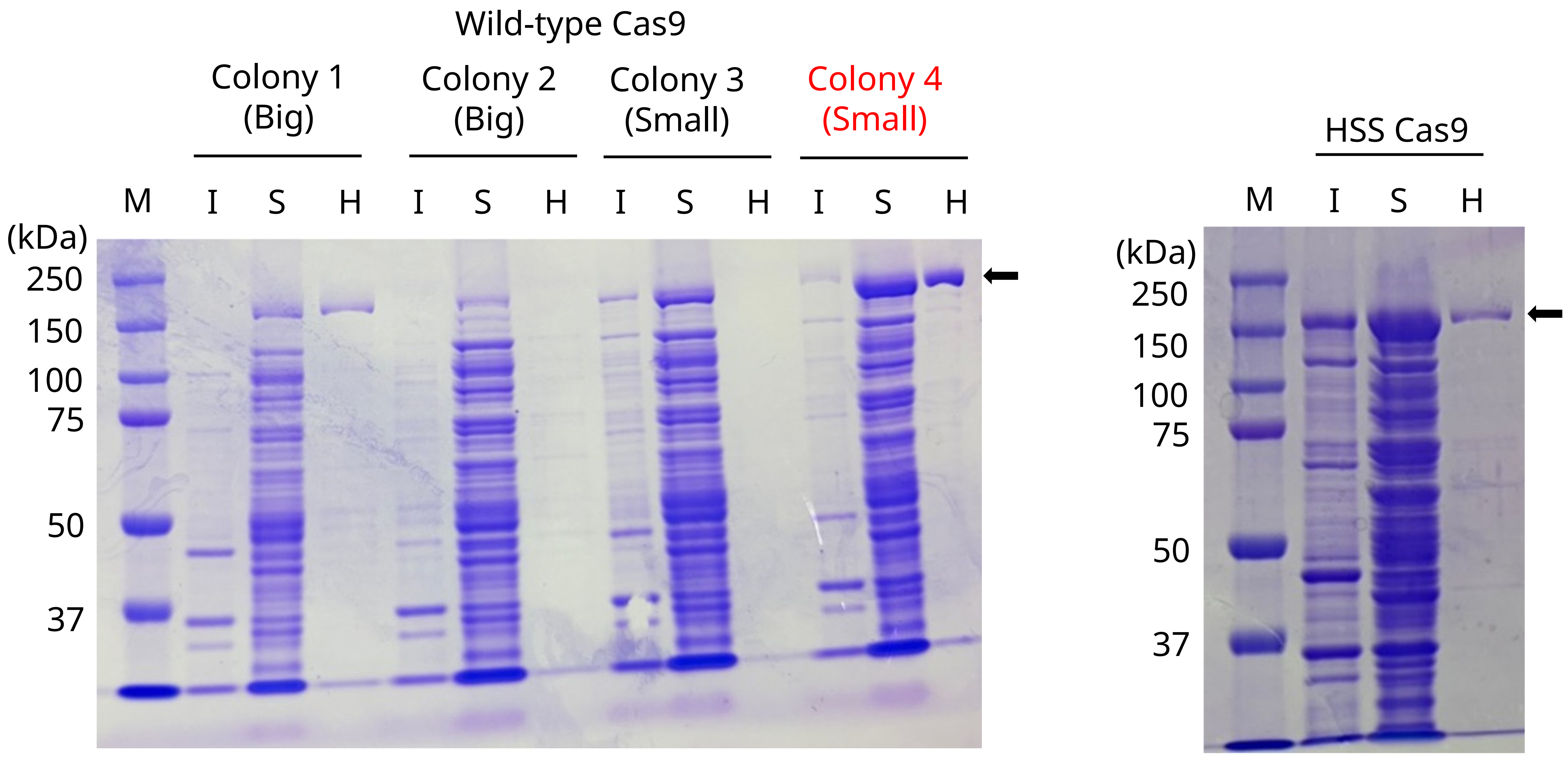

Supplementary Figure 6. CBB stained gel images after SDS-PAGE. “M” shows the marker, “I” shows the insoluble fraction, “S” shows the soluble fraction, and “H” shows the His-tag purified fraction. The black arrow shows the band of recombinant Cas9 protein.

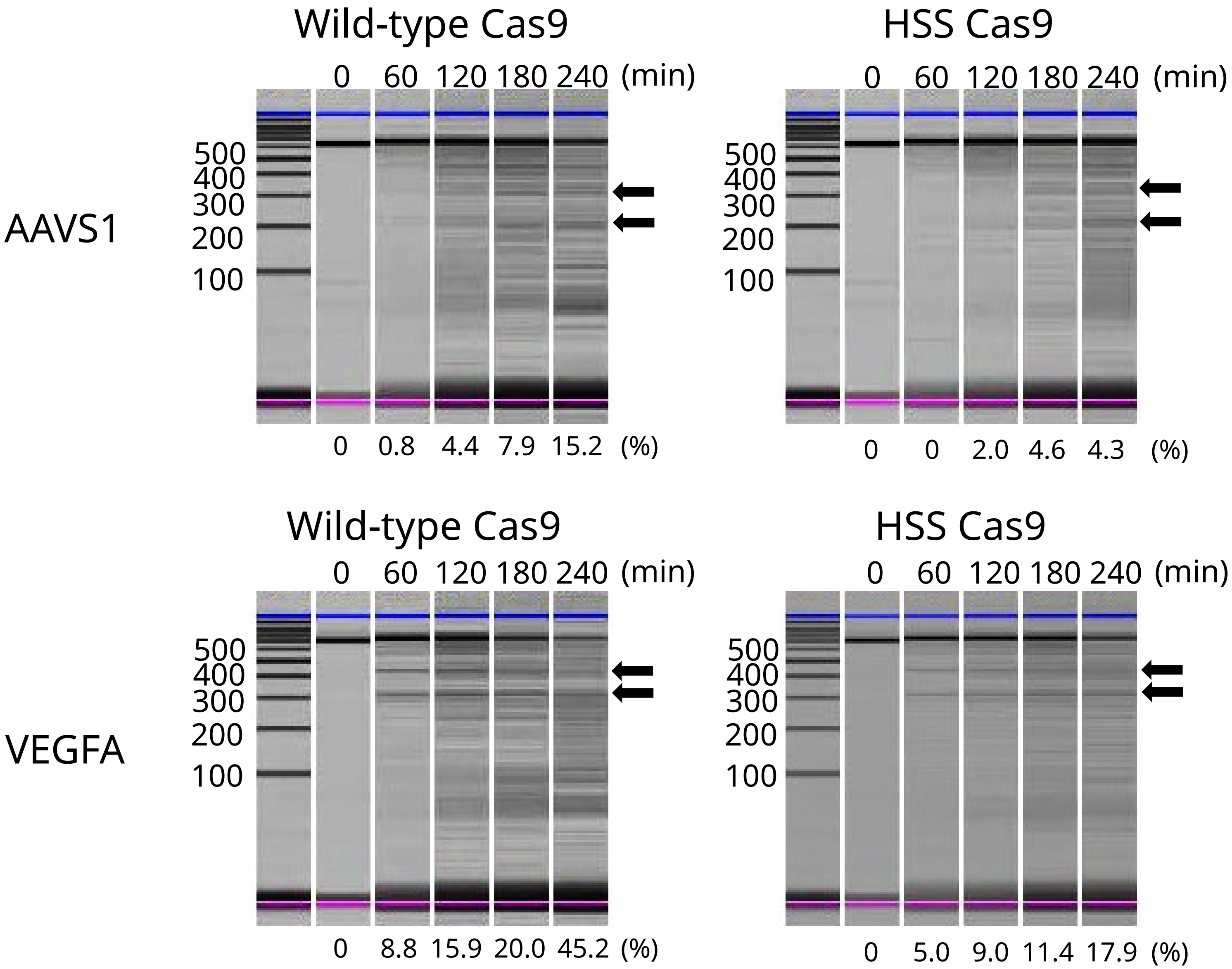

Supplementary Figure 7. In *vitro* cleavage assay of wild-type Cas9 and HSS Cas9. 20 ng of Cas9 and sgRNA were used to cleave 150 ng of PCR-amplified target DNA. Reaction was stopped in 60, 120, 180, and 240 min by degradation of Cas9 using proteinase K. The cleaved fragment were analyzed using MultiNA. Black arrow shows digested fragments.

Supplementary Figure 8. Crystal structure of SpCas9-sgRNA-target DNA complexes.
Each figure is modified from PDB 4UN3, 5F9R, 5Y36, and 7OXA.

A

Before transfection

Wild-type Cas9

Empty vector

HSS Cas9

B

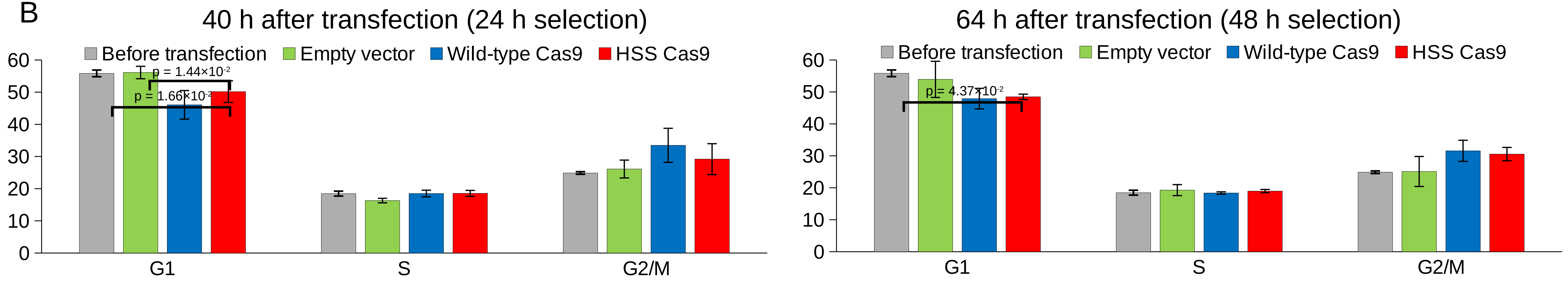

Supplementary Figure 9. Analysis of the Effect of HSS Cas9 on the Cell Cycle. Transfected cells were stained with 5 μg/mL Hoechst 33342. (A) Histogram showing the fluorescence intensity of Hoechst 33342 and the corresponding number of cells. Gate P2 indicates the population of cells in the G1 phase, P3 indicates cells in the S phase, and P4 indicates cells in the G2/M phase. (B) Quantification of the average proportion of cells in each phase of the cell cycle. All pairwise comparisons and their p-values from the Turkey test are summarized in Supplementary Table 17.
